# Identification of core and rare species in metagenome samples based on shotgun metagenomic sequencing, Fourier transforms and spectral comparisons

**DOI:** 10.1038/s43705-021-00010-6

**Published:** 2021-03-24

**Authors:** Marie-Madlen Pust, Burkhard Tümmler

**Affiliations:** 1 Clinic for Paediatric Pneumology, Allergology, and Neonatology, Hannover Medical School (MHH), Hannover, Germany; 2 Biomedical Research in Endstage and Obstructive Lung Disease Hannover (BREATH), German Center for Lung Research, Hannover Medical School, Hannover, Germany

**Keywords:** Metagenomics, Microbial ecology, Next-generation sequencing

## Abstract

In shotgun metagenomic sequencing applications, low signal-to-noise ratios may complicate species-level differentiation of genetically similar core species and impede high-confidence detection of rare species. However, core and rare species can take pivotal roles in their habitats and should hence be studied as one entity to gain insights into the total potential of microbial communities in terms of taxonomy and functionality. Here, we offer a solution towards increased species-level specificity, decreased false discovery and omission rates of core and rare species in complex metagenomic samples by introducing the rare species identifier (raspir) tool. The python software is based on discrete Fourier transforms and spectral comparisons of biological and reference frequency signals obtained from real and ideal distributions of short DNA reads mapping towards circular reference genomes. Simulation-based testing of raspir enabled the detection of rare species with genome coverages of less than 0.2%. Species-level differentiation of rare *Escherichia coli* and *Shigella* spp., as well as the clear delineation between human *Streptococcus* spp. was feasible with low false discovery (1.3%) and omission rates (13%). Publicly available human placenta sequencing data were reanalysed with raspir. Raspir was unable to identify placental microbial communities, reinforcing the sterile womb paradigm.

## Introduction

In shotgun metagenomic sequencing, the total DNA, host and microbial, is extracted from complex biological samples. Random DNA sequencing with reference-based alignment enables the taxonomic identification of bacteria in polymicrobial communities.^1–3^ However, bacteria can often not be discriminated on species-level due to high average nucleotide identities and short genetic sequences that are shared among microbial community members or entries in the reference databases. *Escherichia coli* and *Shigella* spp. for example, are clinically relevant pathogens with distinctive phenotypes but highly similar genotypes. Genetically, they can be assigned to the same species with 16S rRNA gene sequence similarities of >99%.^4–6^ Human airway *Streptococcus* spp. are also genetically closely related and their differentiation remains challenging, e.g., *Streptococcus pneumoniae*, *Streptococcus oralis* and *Streptococcus mitis* exhibit 16S rRNA gene sequence similarities of 99–100%.^7^ So true positive species may be identified by reference-based mapping but misalignments towards homologous sequences of database entries cause dozens to hundreds of false positive hits.^1,8^ Furthermore, even a minimum of DNA contamination may bias the taxonomic interpretation, particularly if the samples were obtained from low-biomass environments.^9–11^ Currently, the problem of false positive species predictions due to misalignments and contamination is slightly attenuated by defining abundance thresholds, where 90–99.9% of the most abundant species (core species) are investigated, whereas the 0.1–10% of the least abundant species (rare species) are discarded.^12–15^ This reduces background noise but comes at the expense of information loss on rare species, which can provide the microbial community with genetic diversity and functional flexibility as well as contribute to human health.^14,16^ In brief, core and rare species take strategic roles in their habitats, but species-level differentiation remains difficult for genetically similar core and the majority of rare species.

Here, we introduce a python tool (*rare species identifier, raspir*) that scans the within-species conservation of the global chromosomal organisation by evaluating the distribution of raw reads mapping towards circular reference genomes. Since gene order is well conserved at the species-level and rapidly lost or extensively clustered as phylogenetic distances increase, it provides a sensitive measure for the differentiation of microbial species.^17^ So, on the hand, if reads align to reference genomes of true positive species, they are expected to spread across the entire genome, despite large gaps in-between the reads in case of low-abundant taxa. On the other hand, if reads are mapping to reference genomes of absent species (false positives), which acquired genes of true positive species, the reads are expected to cluster spatially in the reference genome.^17,18^ Raspir hence distinguishes the uniform read distribution of true positives from the spatial cluster behaviour of false positive species. In addition, structural variants evolve orders of magnitude faster than nucleotide sequence variants and can cause significant phenotypic variations between closely related organisms.^19,20^ Focusing on genome organisation rather than sequence similarity alone, enables raspir to differentiate between genomes with high sequence similarity but different phenotypic behaviour. So, for all pairwise position combinations of short DNA reads aligning to a circular genome, raspir measures the read distances (in base pairs, bp) to generate position-domain signals (Supplementary Text 1). Since raspir considers only the first base position of a read, the tool can be approached for a wide range of DNA insert sizes. Reference position-domain signals are also built with the same number of reads, but with an ideal distribution of reads across the genome (Supplementary Text 1). Biological and reference distance vectors are separately decomposed using the discrete Fourier transform algorithm of NumPy.^21^ Absolute values of Fourier coefficients are used for signal comparisons. Bacterial species are classified as true positives if the reference and biological signals exhibit strong Pearson’s correlations (Correlation coefficient > 0.6, *p* value < 0.05, standard error of estimates < 0.01) and low Euclidean dissimilarity indices (EDI < 0.5).

The applicability of raspir was demonstrated by in-silico simulations of airway microbial communities with *Pseudomonas aeruginosa*, *Rothia mucilaginosa*, *Streptococcus salivarius*, *Eubacterium sulci*, *Streptococcus thermophilus*, *S. pneumoniae*, *S. mitis*, *Streptococcus equinus*, *Staphylococcus aureus* and *E. coli*. *E. coli* was included to evaluate the ability of raspir to differentiate between* E. coli* and *Shigella* spp. Therefore, we generated short (75 bp), single-end DNA reads with the Illumina simulation tool ART (HiSeq 2500).^22^ The number of reads obtained from core species remained constant but increased for rare species during subsequent simulation runs (Supplementary Table 1). Reads were trimmed,^23^ duplicates and low-complexity reads were removed^24^ and the remainder reads were mapped towards a curated reference database of completely sequenced genomes using BWA.^25^ Alignment data (.SAM format) were cleaned with SAMtools, coverage information was obtained^24^ and the final files (.CSV format) were used as input files for raspir. A step-by-step manual is publicly available (see data availability section). For each run (with and without raspir), the number of true positive, true negative, false positive and false negative species was obtained to identify the clinimetric properties specificity, sensitivity, false discovery rate and false omission rate (Supplementary Table 2). Additionally, we downloaded publicly available paired-end Illumina data (HiSeq 2500, 2 ×125 bp, SRA repository: SRP141397) from blank swabs, maternal saliva and placenta samples.^26^ The microbial raw reads were treated as described above. The biological samples were reanalysed with and without raspir.

**Fig. 1:**
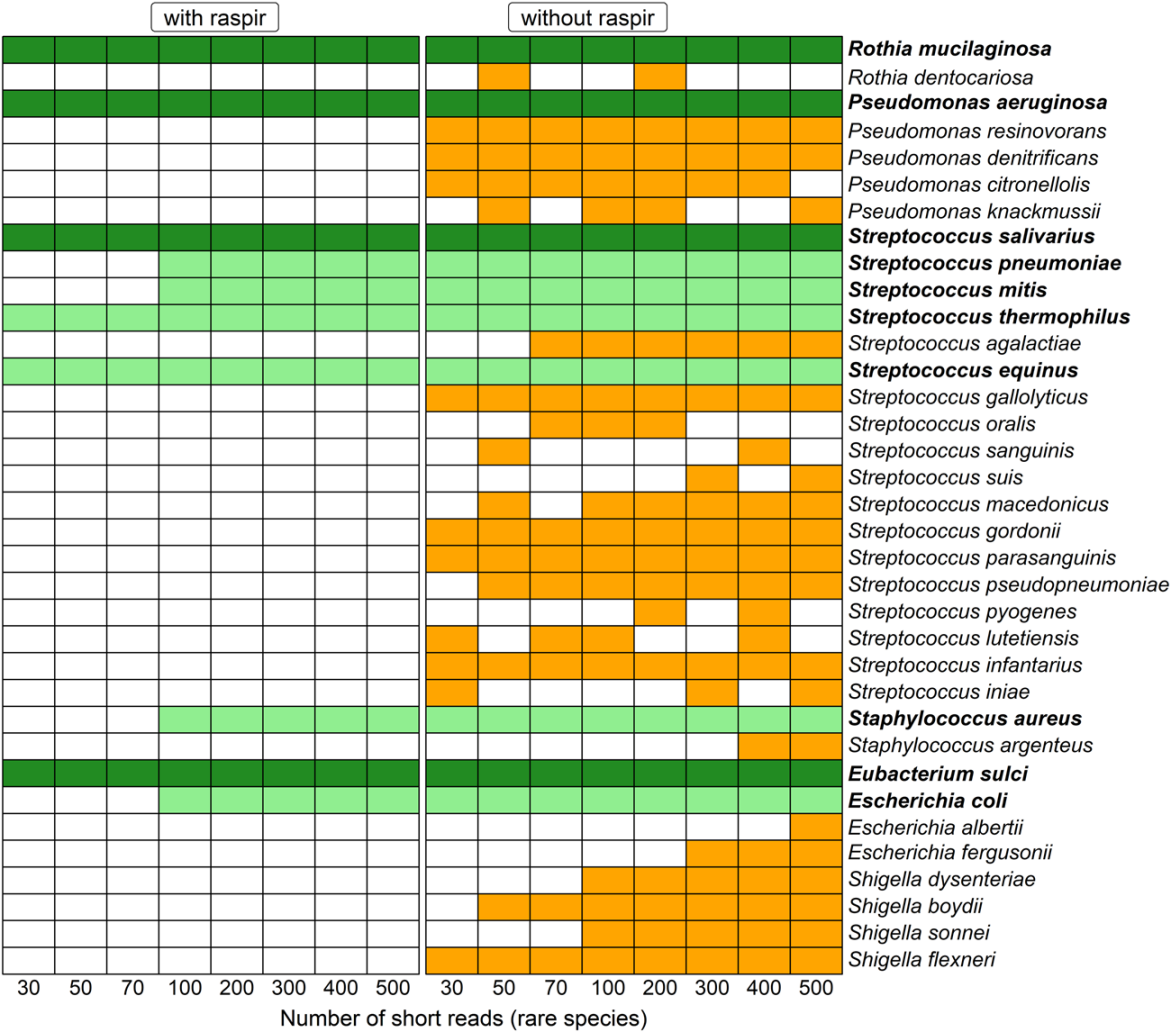
Performance evaluation of raspir on species level based on a representative complete simulation run (seed 222). Bold row names highlight the true positive species of the simulated mock community. The dark-green and light-green colours represent the true positive core and rare species of the community, respectively. The orange colour visualises false positive species. While the read number of the core species remained constant throughout all the runs, the *x*-axis corresponds to the increasing number of short reads (75 bp) that were generated for rare species during the eight simulation runs.

**Fig. 2:**
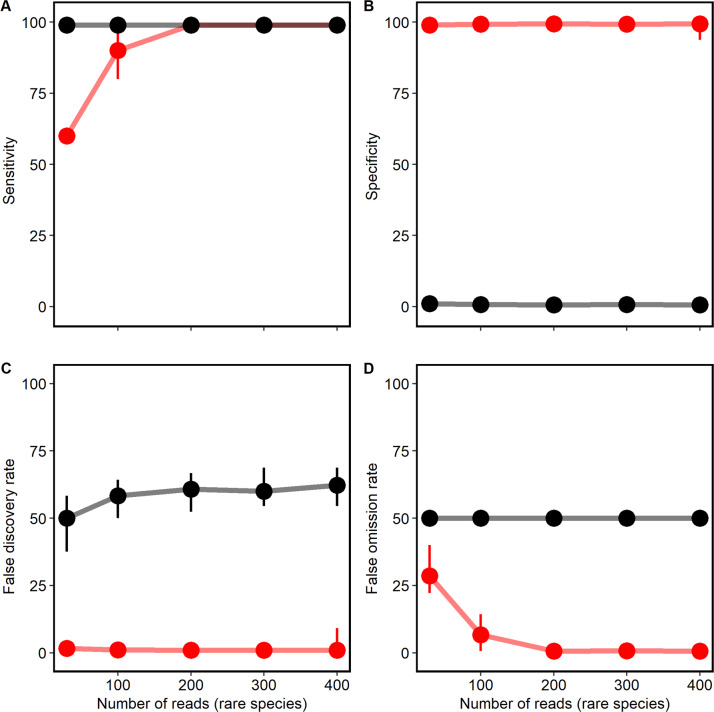
Clinimetric properties of species-level prediction with raspir (red) and without raspir (black). **A** Average test sensitivities of 81.0% and 99.0% were observed for simulation runs with raspir and without raspir, respectively. The test sensitivity was significantly higher without raspir (Mann–Whitney–Wilcoxon, *p* value < 0.0001, effect size *r* = 0.43, confidence intervals = 0.28–0.57). However, the sensitivity was similar for all simulation runs with at least 100 reads per rare species (Mann–Whitney–Wilcoxon, *p* value = 1). **B** Average test specificities of 99.2% and 0.8% were observed for simulation runs with raspir and without raspir, respectively. The specificity with raspir was hence significantly higher (Mann–Whitney–Wilcoxon, *p* value < 0.0001, effect size *r* = 0.87, confidence intervals = 0.86–0.87). **C** Average false discovery rates of 1.3% and 56.7% were observed for simulation runs with raspir and without raspir, respectively. The false discovery rate of raspir was significantly lower (Mann–Whitney–Wilcoxon, *p* value < 0.0001, effect size *r* = 0.87, confidence intervals = 0.87–0.88). **D** Average false omission rates of 12.9% and 50% were observed for simulation runs with raspir and without, respectively. The false omission rate was significantly lower with raspir (Mann–Whitney–Wilcoxon, *p* value < 0.0001, effect size *r* = 0.92, confidence intervals = 0.91–0.94). The points and lines represent median values, the error bars show the minimum and maximum values obtained during all simulations. The individual data points can be obtained from Supplementary Table 2.

During simulation-based testing, raspir reduced the background noise in all runs significantly (Fig. 1). With just 100 short reads of 75 bp lengths, all core and rare species of the mock community were correctly identified as true positives. Considering the range of genome sizes of the rare species in the mock community (Supplementary Table 1), average genome coverages below 0.002 were sufficient for rare species prediction with high specificity and sensitivity. While raspir correctly confirmed the presence of *S. salivarius, S. thermophilus*, *S. pneumoniae*, *S. mitis* and *S. equinus*, false positive *Streptococcus* spp. were discarded (Supplementary Fig. 1). Raspir identified the true positive *E. coli* and dismissed true negative *Escherichia* spp. and *Shigella* spp. (Supplementary Fig. 2). This is a major improvement considering their genetic similarities. Without raspir, *Shigella* spp., various *Escherichia* and *Streptococcus* spp. were falsely predicted to be present (Fig. 1, Supplementary Figs. 1 and 2). Across all simulation runs with twenty different seeds set for the random read generator, we found that incorporating raspir into the workflow let initially to a lower test sensitivity for rare species with less than 100 raw reads (Fig. 2A), in contrast to the test specificity, which remained on average by 98%. (Fig. 2B). In consideration of the prevalence; however, raspir achieved a significant decline in both false discovery (Fig. 2C) and false omission rates (Fig. 2D) by approximately 55% and 37% at all times, respectively.

Next, we approached publicly available real-world datasets to illustrate the value of raspir for answering critical questions of principal biological relevance. In recent years, it has been reported that the healthy placenta harbours a distinct microbiome, suggesting that the foetus comes into contact with commensal bacteria from early on.^27^ However, several follow-up studies were unable to reproduce a placenta-specific microbial signal from this low-biomass environment, indicating that the heathy foetal environment is sterile.^26,28^ This includes the study of Leiby et al., who applied shotgun sequencing to human placenta samples, maternal saliva and controls.^26^ While they recovered a small proportion of microbial reads from placenta samples, the microbial community composition was not distinguishable from negative controls. However, some placenta samples contained more *Vibrio* bacteria than negative controls but *Vibrio* spp. were artificially spiked into positive controls, indicating that barcode misreading was responsible for the Vibrio detection.^26^ Our reanalysis of these datasets with raspir confirmed the complete absence of placental microbial communities (Supplementary Fig. 3), reinforcing the sterile womb paradigm.^26,28^ Raspir solely recovered the well-known laboratory contaminant *Ralstonia pickettii* from placenta samples, which is commonly isolated from various pharmaceutical reagents and equipment, including laboratory-based purified water systems.^29^ Low-abundant *R. pickettii* was also detected in all maternal saliva and negative controls by raspir, irrespectively of the sample’s sequencing depths or the number of *R. pickettii*—specific raw reads (Supplementary Fig. 4).

We subsequently analysed the maternal saliva samples of the study^26^ and compared the inter-patient weighted Jaccard distances^30^ in microbial community composition obtained without raspir (black, Supplementary Fig. 5) with the intra-patient distances obtained with versus without raspir (green, Supplementary Fig. 5). For the core species (Supplementary Fig. 5A), inter-patient distances of microbial community composition (black) were significantly larger than intra-patient distances (green). Therefore, patient-specific signatures of core microbial communities were reliably identified with and without raspir. This is an encouraging outcome, considering that most shotgun metagenomic sequencing studies remove low-abundant taxa from downstream analyses. However, for the rare species community (Supplementary Fig. 5B), significantly higher dissimilarity scores were obtained for intra-patient (green) compared to inter-patient (black) microbial communities, indicating that raspir is particularly effective for investigating the rare species of complex communities with high confidence.

In conclusion, raspir is based on discrete Fourier transforms of read position signals and identifies core and rare species with low false discovery and omission rates. The tool can be integrated into standard workflows and may hence be a valuable addition to metagenomic pipelines in future applications.

## Supplementary information

Supplementary Table 1

Supplementary Table 2

Supplementary Figure 1

Supplementary Figure 2

Supplementary Figure 3

Supplementary Figure 4

Supplementary Figure 5

Supplementary Text 1

Supplementary legends

## Data Availability

The manual, reference database and python code of raspir are available from https://github.com/mmpust/raspir. R and bash scripts for the performance evaluation can be obtained from https://github.com/mmpust/raspir_evaluation.
