# Supplementary material for: Identification of core and rare species in metagenome samples based on shotgun metagenomic sequencing, Fourier transforms and spectral comparisons
Source: ISME Commun. 2021 Mar 24;1:2. doi: 10.1038/s43705-021-00010-6 (PMC9645229; doi:10.1038/s43705-021-00010-6)
Supplement: Supplementary file 1 — Supplementary Table 1 [file 43705_2021_10_MOESM1_ESM.docx]

| **Supplementary Table 1. Selected species and number of reads per species for eight simulation runs.** Simulation runs (1-8) were repeated with different seeds set for the ART Illumina read generator. Results for individual runs can be obtained from Supplementary Table 02. | | | | | | | | | |
| --- | --- | --- | --- | --- | --- | --- | --- | --- | --- |
|  | | **Run1** | **Run2** | **Run3** | **Run4** | **Run5** | **Run6** | **Run7** | **Run8** |
| **Core species** | **Genome size (in bp)** | **Number of reads** | **Number of reads** | **Number of reads** | **Number of reads** | **Number of reads** | **Number of reads** | **Number of reads** | **Number of reads** |
| *Streptococcus salivarius* | 2 210 574 | 20000 | 20000 | 20000 | 20000 | 20000 | 20000 | 20000 | 20000 |
| *Rothia mucilaginosa* | 2 264 603 | 8000 | 8000 | 8000 | 8000 | 8000 | 8000 | 8000 | 8000 |
| *Pseudomonas aeruginosa* | 6 264 404 | 10000 | 10000 | 10000 | 10000 | 10000 | 10000 | 10000 | 10000 |
| *Eubacterium sulci* | 1 739 380 | 10000 | 10000 | 10000 | 10000 | 10000 | 10000 | 10000 | 10000 |
| Total reads |  | 48000 | 48000 | 48000 | 48000 | 48000 | 48000 | 48000 | 48000 |
| Relative abundance (in %) |  | 99.6 | 99.4 | 99.1 | 98.8 | 97.6 | 96.4 | 95.2 | 94.1 |
| **Rare species** |  |  |  |  |  |  |  |  |  |
| *Streptococcus thermophilus* | 1 796 846 | 30 | 50 | 70 | 100 | 200 | 300 | 400 | 500 |
| *Streptococcus pneumoniae* | 2 160 842 | 30 | 50 | 70 | 100 | 200 | 300 | 400 | 500 |
| *Streptococcus mitis* | 2 146 611 | 30 | 50 | 70 | 100 | 200 | 300 | 400 | 500 |
| *Streptococcus equinus* | 2 074 978 | 30 | 50 | 70 | 100 | 200 | 300 | 400 | 500 |
| *Staphylococcus aureus* | 2 742 531 | 30 | 50 | 70 | 100 | 200 | 300 | 400 | 500 |
| *Escherichia coli* | 4 641 652 | 30 | 50 | 70 | 100 | 200 | 300 | 400 | 500 |
| Total reads |  | 180 | 300 | 420 | 600 | 1200 | 1800 | 2400 | 3000 |
| Relative abundance (in %) |  | 0.4 | 0.6 | 0.9 | 1.2 | 2.4 | 3.6 | 4.8 | 5.9 |
