# Supplementary material for: Identification of core and rare species in metagenome samples based on shotgun metagenomic sequencing, Fourier transforms and spectral comparisons
Source: ISME Commun. 2021 Mar 24;1:2. doi: 10.1038/s43705-021-00010-6 (PMC9645229; doi:10.1038/s43705-021-00010-6)
Supplement: Supplementary file 2 — Supplementary Table 2 [file 43705_2021_10_MOESM2_ESM.docx]

| **Supplementary table 2. Final result of the eight simulation runs** with either 30, 50, 70, 100, 200, 300, 400 and 500 reads for the rare species. The eight simulation runs were repeated with twenty different seeds and with two aligner tools (bowtie2 and bwa). The table shows raw count data for true positive (TP), false positive (FP), true negative (TN), false negative (FN) species. The specificity (true negative/(true negative + false positive)) and sensitivity (true positive / (true positive + false negative)), as well as the false discovery rate (FDR, 1 – (true positive / (true positive + false positive)) and the false omission rate (FOR, 1 – (true negative / (true negative + false negative)) are given as percentage values. | | | | | | | | | | | | | | | | | | |
| --- | --- | --- | --- | --- | --- | --- | --- | --- | --- | --- | --- | --- | --- | --- | --- | --- | --- | --- |
| Variables | | | With raspir | | | | | | | | Without raspir | | | | | | | |
| Count | Seed | Aligner | TP | FP | TN | FN | Specificity | Sensitivity | FDR | FOR | TP | FP | TN | FN | Specificity | Sensitivity | FDR | FOR |
| 30 | 202 | bowtie2 | 6 | 0.1 | 9 | 4 | 98.9 | 60 | 1.6 | 30.8 | 10 | 9 | 0.1 | 0.1 | 1.1 | 99 | 47.4 | 50 |
| 30 | 202 | bwa | 6 | 0.1 | 13 | 4 | 99.2 | 60 | 1.6 | 23.5 | 10 | 13 | 0.1 | 0.1 | 0.8 | 99 | 56.5 | 50 |
| 30 | 203 | bowtie2 | 6 | 0.1 | 9 | 4 | 98.9 | 60 | 1.6 | 30.8 | 10 | 9 | 0.1 | 0.1 | 1.1 | 99 | 47.4 | 50 |
| 30 | 203 | bwa | 6 | 0.1 | 12 | 4 | 99.2 | 60 | 1.6 | 25 | 10 | 12 | 0.1 | 0.1 | 0.8 | 99 | 54.5 | 50 |
| 30 | 204 | bowtie2 | 6 | 0.1 | 10 | 4 | 99 | 60 | 1.6 | 28.6 | 10 | 10 | 0.1 | 0.1 | 1 | 99 | 50 | 50 |
| 30 | 204 | bwa | 6 | 0.1 | 14 | 4 | 99.3 | 60 | 1.6 | 22.2 | 10 | 14 | 0.1 | 0.1 | 0.7 | 99 | 58.3 | 50 |
| 30 | 205 | bowtie2 | 6 | 0.1 | 9 | 4 | 98.9 | 60 | 1.6 | 30.8 | 10 | 9 | 0.1 | 0.1 | 1.1 | 99 | 47.4 | 50 |
| 30 | 205 | bwa | 6 | 0.1 | 11 | 4 | 99.1 | 60 | 1.6 | 26.7 | 10 | 11 | 0.1 | 0.1 | 0.9 | 99 | 52.4 | 50 |
| 30 | 206 | bowtie2 | 6 | 0.1 | 13 | 4 | 99.2 | 60 | 1.6 | 23.5 | 10 | 13 | 0.1 | 0.1 | 0.8 | 99 | 56.5 | 50 |
| 30 | 206 | bwa | 6 | 0.1 | 11 | 4 | 99.1 | 60 | 1.6 | 26.7 | 10 | 11 | 0.1 | 0.1 | 0.9 | 99 | 52.4 | 50 |
| 30 | 207 | bowtie2 | 6 | 0.1 | 10 | 4 | 99 | 60 | 1.6 | 28.6 | 10 | 10 | 0.1 | 0.1 | 1 | 99 | 50 | 50 |
| 30 | 207 | bwa | 6 | 0.1 | 7 | 4 | 98.6 | 60 | 1.6 | 36.4 | 10 | 7 | 0.1 | 0.1 | 1.4 | 99 | 41.2 | 50 |
| 30 | 208 | bowtie2 | 6 | 0.1 | 7 | 4 | 98.6 | 60 | 1.6 | 36.4 | 10 | 7 | 0.1 | 0.1 | 1.4 | 99 | 41.2 | 50 |
| 30 | 208 | bwa | 6 | 0.1 | 12 | 4 | 99.2 | 60 | 1.6 | 25 | 10 | 12 | 0.1 | 0.1 | 0.8 | 99 | 54.5 | 50 |
| 30 | 209 | bowtie2 | 6 | 0.1 | 9 | 4 | 98.9 | 60 | 1.6 | 30.8 | 10 | 9 | 0.1 | 0.1 | 1.1 | 99 | 47.4 | 50 |
| 30 | 209 | bwa | 6 | 0.1 | 14 | 4 | 99.3 | 60 | 1.6 | 22.2 | 10 | 14 | 0.1 | 0.1 | 0.7 | 99 | 58.3 | 50 |
| 30 | 210 | bowtie2 | 6 | 0.1 | 8 | 4 | 98.8 | 60 | 1.6 | 33.3 | 10 | 8 | 0.1 | 0.1 | 1.2 | 99 | 44.4 | 50 |
| 30 | 210 | bwa | 6 | 0.1 | 9 | 4 | 98.9 | 60 | 1.6 | 30.8 | 10 | 9 | 0.1 | 0.1 | 1.1 | 99 | 47.4 | 50 |
| 30 | 211 | bowtie2 | 6 | 0.1 | 8 | 4 | 98.8 | 60 | 1.6 | 33.3 | 10 | 8 | 0.1 | 0.1 | 1.2 | 99 | 44.4 | 50 |
| 30 | 211 | bwa | 6 | 0.1 | 6 | 4 | 98.4 | 60 | 1.6 | 40 | 10 | 6 | 0.1 | 0.1 | 1.6 | 99 | 37.5 | 50 |
| 30 | 212 | bowtie2 | 6 | 0.1 | 6 | 4 | 98.4 | 60 | 1.6 | 40 | 10 | 6 | 0.1 | 0.1 | 1.6 | 99 | 37.5 | 50 |
| 30 | 212 | bwa | 6 | 0.1 | 9 | 4 | 98.9 | 60 | 1.6 | 30.8 | 10 | 9 | 0.1 | 0.1 | 1.1 | 99 | 47.4 | 50 |
| 30 | 213 | bowtie2 | 6 | 0.1 | 8 | 4 | 98.8 | 60 | 1.6 | 33.3 | 10 | 8 | 0.1 | 0.1 | 1.2 | 99 | 44.4 | 50 |
| 30 | 213 | bwa | 6 | 0.1 | 11 | 4 | 99.1 | 60 | 1.6 | 26.7 | 10 | 11 | 0.1 | 0.1 | 0.9 | 99 | 52.4 | 50 |
| 30 | 214 | bowtie2 | 6 | 0.1 | 7 | 4 | 98.6 | 60 | 1.6 | 36.4 | 10 | 7 | 0.1 | 0.1 | 1.4 | 99 | 41.2 | 50 |
| 30 | 214 | bwa | 6 | 0.1 | 11 | 4 | 99.1 | 60 | 1.6 | 26.7 | 10 | 11 | 0.1 | 0.1 | 0.9 | 99 | 52.4 | 50 |
| 30 | 215 | bowtie2 | 6 | 0.1 | 9 | 4 | 98.9 | 60 | 1.6 | 30.8 | 10 | 9 | 0.1 | 0.1 | 1.1 | 99 | 47.4 | 50 |
| 30 | 215 | bwa | 6 | 0.1 | 10 | 4 | 99 | 60 | 1.6 | 28.6 | 10 | 10 | 0.1 | 0.1 | 1 | 99 | 50 | 50 |
| 30 | 216 | bowtie2 | 6 | 0.1 | 11 | 4 | 99.1 | 60 | 1.6 | 26.7 | 10 | 11 | 0.1 | 0.1 | 0.9 | 99 | 52.4 | 50 |
| 30 | 216 | bwa | 6 | 0.1 | 10 | 4 | 99 | 60 | 1.6 | 28.6 | 10 | 10 | 0.1 | 0.1 | 1 | 99 | 50 | 50 |
| 30 | 217 | bowtie2 | 6 | 0.1 | 11 | 4 | 99.1 | 60 | 1.6 | 26.7 | 10 | 11 | 0.1 | 0.1 | 0.9 | 99 | 52.4 | 50 |
| 30 | 217 | bwa | 6 | 0.1 | 12 | 4 | 99.2 | 60 | 1.6 | 25 | 10 | 12 | 0.1 | 0.1 | 0.8 | 99 | 54.5 | 50 |
| 30 | 218 | bowtie2 | 6 | 0.1 | 8 | 4 | 98.8 | 60 | 1.6 | 33.3 | 10 | 8 | 0.1 | 0.1 | 1.2 | 99 | 44.4 | 50 |
| 30 | 218 | bwa | 6 | 0.1 | 10 | 4 | 99 | 60 | 1.6 | 28.6 | 10 | 10 | 0.1 | 0.1 | 1 | 99 | 50 | 50 |
| 30 | 219 | bowtie2 | 6 | 0.1 | 9 | 4 | 98.9 | 60 | 1.6 | 30.8 | 10 | 9 | 0.1 | 0.1 | 1.1 | 99 | 47.4 | 50 |
| 30 | 219 | bwa | 6 | 0.1 | 8 | 4 | 98.8 | 60 | 1.6 | 33.3 | 10 | 8 | 0.1 | 0.1 | 1.2 | 99 | 44.4 | 50 |
| 30 | 220 | bowtie2 | 6 | 0.1 | 7 | 4 | 98.6 | 60 | 1.6 | 36.4 | 10 | 7 | 0.1 | 0.1 | 1.4 | 99 | 41.2 | 50 |
| 30 | 220 | bwa | 6 | 0.1 | 12 | 4 | 99.2 | 60 | 1.6 | 25 | 10 | 12 | 0.1 | 0.1 | 0.8 | 99 | 54.5 | 50 |
| 30 | 221 | bowtie2 | 6 | 0.1 | 10 | 4 | 99 | 60 | 1.6 | 28.6 | 10 | 10 | 0.1 | 0.1 | 1 | 99 | 50 | 50 |
| 30 | 221 | bwa | 6 | 0.1 | 10 | 4 | 99 | 60 | 1.6 | 28.6 | 10 | 10 | 0.1 | 0.1 | 1 | 99 | 50 | 50 |
| 30 | 222 | bowtie2 | 6 | 0.1 | 8 | 4 | 98.8 | 60 | 1.6 | 33.3 | 10 | 8 | 0.1 | 0.1 | 1.2 | 99 | 44.4 | 50 |
| 30 | 222 | bwa | 6 | 0.1 | 10 | 4 | 99 | 60 | 1.6 | 28.6 | 10 | 10 | 0.1 | 0.1 | 1 | 99 | 50 | 50 |
| 50 | 202 | bowtie2 | 6 | 0.1 | 9 | 4 | 98.9 | 60 | 1.6 | 30.8 | 10 | 9 | 0.1 | 0.1 | 1.1 | 99 | 47.4 | 50 |
| 50 | 202 | bwa | 6 | 0.1 | 14 | 4 | 99.3 | 60 | 1.6 | 22.2 | 10 | 14 | 0.1 | 0.1 | 0.7 | 99 | 58.3 | 50 |
| 50 | 203 | bowtie2 | 6 | 0.1 | 10 | 4 | 99 | 60 | 1.6 | 28.6 | 10 | 10 | 0.1 | 0.1 | 1 | 99 | 50 | 50 |
| 50 | 203 | bwa | 6 | 0.1 | 10 | 4 | 99 | 60 | 1.6 | 28.6 | 10 | 10 | 0.1 | 0.1 | 1 | 99 | 50 | 50 |
| 50 | 204 | bowtie2 | 6 | 0.1 | 12 | 4 | 99.2 | 60 | 1.6 | 25 | 10 | 12 | 0.1 | 0.1 | 0.8 | 99 | 54.5 | 50 |
| 50 | 204 | bwa | 6 | 0.1 | 13 | 4 | 99.2 | 60 | 1.6 | 23.5 | 10 | 13 | 0.1 | 0.1 | 0.8 | 99 | 56.5 | 50 |
| 50 | 205 | bowtie2 | 6 | 0.1 | 10 | 4 | 99 | 60 | 1.6 | 28.6 | 10 | 10 | 0.1 | 0.1 | 1 | 99 | 50 | 50 |
| 50 | 205 | bwa | 6 | 0.1 | 9 | 4 | 98.9 | 60 | 1.6 | 30.8 | 10 | 9 | 0.1 | 0.1 | 1.1 | 99 | 47.4 | 50 |
| 50 | 206 | bowtie2 | 6 | 0.1 | 15 | 4 | 99.3 | 60 | 1.6 | 21.1 | 10 | 15 | 0.1 | 0.1 | 0.7 | 99 | 60 | 50 |
| 50 | 206 | bwa | 6 | 0.1 | 12 | 4 | 99.2 | 60 | 1.6 | 25 | 10 | 12 | 0.1 | 0.1 | 0.8 | 99 | 54.5 | 50 |
| 50 | 207 | bowtie2 | 6 | 0.1 | 10 | 4 | 99 | 60 | 1.6 | 28.6 | 10 | 10 | 0.1 | 0.1 | 1 | 99 | 50 | 50 |
| 50 | 207 | bwa | 6 | 0.1 | 12 | 4 | 99.2 | 60 | 1.6 | 25 | 10 | 12 | 0.1 | 0.1 | 0.8 | 99 | 54.5 | 50 |
| 50 | 208 | bowtie2 | 6 | 0.1 | 8 | 4 | 98.8 | 60 | 1.6 | 33.3 | 10 | 8 | 0.1 | 0.1 | 1.2 | 99 | 44.4 | 50 |
| 50 | 208 | bwa | 6 | 0.1 | 9 | 4 | 98.9 | 60 | 1.6 | 30.8 | 10 | 9 | 0.1 | 0.1 | 1.1 | 99 | 47.4 | 50 |
| 50 | 209 | bowtie2 | 6 | 0.1 | 10 | 4 | 99 | 60 | 1.6 | 28.6 | 10 | 10 | 0.1 | 0.1 | 1 | 99 | 50 | 50 |
| 50 | 209 | bwa | 6 | 0.1 | 15 | 4 | 99.3 | 60 | 1.6 | 21.1 | 10 | 15 | 0.1 | 0.1 | 0.7 | 99 | 60 | 50 |
| 50 | 210 | bowtie2 | 6 | 0.1 | 11 | 4 | 99.1 | 60 | 1.6 | 26.7 | 10 | 11 | 0.1 | 0.1 | 0.9 | 99 | 52.4 | 50 |
| 50 | 210 | bwa | 6 | 0.1 | 11 | 4 | 99.1 | 60 | 1.6 | 26.7 | 10 | 11 | 0.1 | 0.1 | 0.9 | 99 | 52.4 | 50 |
| 50 | 211 | bowtie2 | 6 | 0.1 | 8 | 4 | 98.8 | 60 | 1.6 | 33.3 | 10 | 8 | 0.1 | 0.1 | 1.2 | 99 | 44.4 | 50 |
| 50 | 211 | bwa | 6 | 0.1 | 14 | 4 | 99.3 | 60 | 1.6 | 22.2 | 10 | 14 | 0.1 | 0.1 | 0.7 | 99 | 58.3 | 50 |
| 50 | 212 | bowtie2 | 6 | 0.1 | 7 | 4 | 98.6 | 60 | 1.6 | 36.4 | 10 | 7 | 0.1 | 0.1 | 1.4 | 99 | 41.2 | 50 |
| 50 | 212 | bwa | 6 | 0.1 | 12 | 4 | 99.2 | 60 | 1.6 | 25 | 10 | 12 | 0.1 | 0.1 | 0.8 | 99 | 54.5 | 50 |
| 50 | 213 | bowtie2 | 6 | 0.1 | 11 | 4 | 99.1 | 60 | 1.6 | 26.7 | 10 | 11 | 0.1 | 0.1 | 0.9 | 99 | 52.4 | 50 |
| 50 | 213 | bwa | 6 | 0.1 | 13 | 4 | 99.2 | 60 | 1.6 | 23.5 | 10 | 13 | 0.1 | 0.1 | 0.8 | 99 | 56.5 | 50 |
| 50 | 214 | bowtie2 | 6 | 0.1 | 10 | 4 | 99 | 60 | 1.6 | 28.6 | 10 | 10 | 0.1 | 0.1 | 1 | 99 | 50 | 50 |
| 50 | 214 | bwa | 6 | 0.1 | 15 | 4 | 99.3 | 60 | 1.6 | 21.1 | 10 | 15 | 0.1 | 0.1 | 0.7 | 99 | 60 | 50 |
| 50 | 215 | bowtie2 | 6 | 0.1 | 11 | 4 | 99.1 | 60 | 1.6 | 26.7 | 10 | 11 | 0.1 | 0.1 | 0.9 | 99 | 52.4 | 50 |
| 50 | 215 | bwa | 6 | 0.1 | 11 | 4 | 99.1 | 60 | 1.6 | 26.7 | 10 | 11 | 0.1 | 0.1 | 0.9 | 99 | 52.4 | 50 |
| 50 | 216 | bowtie2 | 6 | 0.1 | 10 | 4 | 99 | 60 | 1.6 | 28.6 | 10 | 10 | 0.1 | 0.1 | 1 | 99 | 50 | 50 |
| 50 | 216 | bwa | 6 | 0.1 | 14 | 4 | 99.3 | 60 | 1.6 | 22.2 | 10 | 14 | 0.1 | 0.1 | 0.7 | 99 | 58.3 | 50 |
| 50 | 217 | bowtie2 | 6 | 0.1 | 11 | 4 | 99.1 | 60 | 1.6 | 26.7 | 10 | 11 | 0.1 | 0.1 | 0.9 | 99 | 52.4 | 50 |
| 50 | 217 | bwa | 6 | 0.1 | 12 | 4 | 99.2 | 60 | 1.6 | 25 | 10 | 12 | 0.1 | 0.1 | 0.8 | 99 | 54.5 | 50 |
| 50 | 218 | bowtie2 | 6 | 0.1 | 10 | 4 | 99 | 60 | 1.6 | 28.6 | 10 | 10 | 0.1 | 0.1 | 1 | 99 | 50 | 50 |
| 50 | 218 | bwa | 6 | 0.1 | 15 | 4 | 99.3 | 60 | 1.6 | 21.1 | 10 | 15 | 0.1 | 0.1 | 0.7 | 99 | 60 | 50 |
| 50 | 219 | bowtie2 | 6 | 0.1 | 11 | 4 | 99.1 | 60 | 1.6 | 26.7 | 10 | 11 | 0.1 | 0.1 | 0.9 | 99 | 52.4 | 50 |
| 50 | 219 | bwa | 6 | 0.1 | 9 | 4 | 98.9 | 60 | 1.6 | 30.8 | 10 | 9 | 0.1 | 0.1 | 1.1 | 99 | 47.4 | 50 |
| 50 | 220 | bowtie2 | 6 | 0.1 | 7 | 4 | 98.6 | 60 | 1.6 | 36.4 | 10 | 7 | 0.1 | 0.1 | 1.4 | 99 | 41.2 | 50 |
| 50 | 220 | bwa | 6 | 0.1 | 12 | 4 | 99.2 | 60 | 1.6 | 25 | 10 | 12 | 0.1 | 0.1 | 0.8 | 99 | 54.5 | 50 |
| 50 | 221 | bowtie2 | 6 | 0.1 | 14 | 4 | 99.3 | 60 | 1.6 | 22.2 | 10 | 14 | 0.1 | 0.1 | 0.7 | 99 | 58.3 | 50 |
| 50 | 221 | bwa | 6 | 0.1 | 10 | 4 | 99 | 60 | 1.6 | 28.6 | 10 | 10 | 0.1 | 0.1 | 1 | 99 | 50 | 50 |
| 50 | 222 | bowtie2 | 6 | 0.1 | 9 | 4 | 98.9 | 60 | 1.6 | 30.8 | 10 | 9 | 0.1 | 0.1 | 1.1 | 99 | 47.4 | 50 |
| 50 | 222 | bwa | 6 | 0.1 | 13 | 4 | 99.2 | 60 | 1.6 | 23.5 | 10 | 13 | 0.1 | 0.1 | 0.8 | 99 | 56.5 | 50 |
| 70 | 202 | bowtie2 | 6 | 0.1 | 12 | 4 | 99.2 | 60 | 1.6 | 25 | 10 | 12 | 0.1 | 0.1 | 0.8 | 99 | 54.5 | 50 |
| 70 | 202 | bwa | 6 | 0.1 | 14 | 4 | 99.3 | 60 | 1.6 | 22.2 | 10 | 14 | 0.1 | 0.1 | 0.7 | 99 | 58.3 | 50 |
| 70 | 203 | bowtie2 | 6 | 0.1 | 13 | 4 | 99.2 | 60 | 1.6 | 23.5 | 10 | 13 | 0.1 | 0.1 | 0.8 | 99 | 56.5 | 50 |
| 70 | 203 | bwa | 6 | 0.1 | 16 | 4 | 99.4 | 60 | 1.6 | 20 | 10 | 16 | 0.1 | 0.1 | 0.6 | 99 | 61.5 | 50 |
| 70 | 204 | bowtie2 | 6 | 0.1 | 12 | 4 | 99.2 | 60 | 1.6 | 25 | 10 | 12 | 0.1 | 0.1 | 0.8 | 99 | 54.5 | 50 |
| 70 | 204 | bwa | 6 | 0.1 | 15 | 4 | 99.3 | 60 | 1.6 | 21.1 | 10 | 15 | 0.1 | 0.1 | 0.7 | 99 | 60 | 50 |
| 70 | 205 | bowtie2 | 6 | 0.1 | 11 | 4 | 99.1 | 60 | 1.6 | 26.7 | 10 | 11 | 0.1 | 0.1 | 0.9 | 99 | 52.4 | 50 |
| 70 | 205 | bwa | 6 | 0.1 | 14 | 4 | 99.3 | 60 | 1.6 | 22.2 | 10 | 14 | 0.1 | 0.1 | 0.7 | 99 | 58.3 | 50 |
| 70 | 206 | bowtie2 | 6 | 0.1 | 16 | 4 | 99.4 | 60 | 1.6 | 20 | 10 | 16 | 0.1 | 0.1 | 0.6 | 99 | 61.5 | 50 |
| 70 | 206 | bwa | 6 | 0.1 | 14 | 4 | 99.3 | 60 | 1.6 | 22.2 | 10 | 14 | 0.1 | 0.1 | 0.7 | 99 | 58.3 | 50 |
| 70 | 207 | bowtie2 | 6 | 0.1 | 10 | 4 | 99 | 60 | 1.6 | 28.6 | 10 | 10 | 0.1 | 0.1 | 1 | 99 | 50 | 50 |
| 70 | 207 | bwa | 6 | 0.1 | 14 | 4 | 99.3 | 60 | 1.6 | 22.2 | 10 | 14 | 0.1 | 0.1 | 0.7 | 99 | 58.3 | 50 |
| 70 | 208 | bowtie2 | 6 | 0.1 | 10 | 4 | 99 | 60 | 1.6 | 28.6 | 10 | 10 | 0.1 | 0.1 | 1 | 99 | 50 | 50 |
| 70 | 208 | bwa | 6 | 0.1 | 11 | 4 | 99.1 | 60 | 1.6 | 26.7 | 10 | 11 | 0.1 | 0.1 | 0.9 | 99 | 52.4 | 50 |
| 70 | 209 | bowtie2 | 6 | 0.1 | 11 | 4 | 99.1 | 60 | 1.6 | 26.7 | 10 | 11 | 0.1 | 0.1 | 0.9 | 99 | 52.4 | 50 |
| 70 | 209 | bwa | 6 | 0.1 | 17 | 4 | 99.4 | 60 | 1.6 | 19 | 10 | 17 | 0.1 | 0.1 | 0.6 | 99 | 63 | 50 |
| 70 | 210 | bowtie2 | 6 | 0.1 | 12 | 4 | 99.2 | 60 | 1.6 | 25 | 10 | 12 | 0.1 | 0.1 | 0.8 | 99 | 54.5 | 50 |
| 70 | 210 | bwa | 6 | 0.1 | 15 | 4 | 99.3 | 60 | 1.6 | 21.1 | 10 | 15 | 0.1 | 0.1 | 0.7 | 99 | 60 | 50 |
| 70 | 211 | bowtie2 | 6 | 0.1 | 11 | 4 | 99.1 | 60 | 1.6 | 26.7 | 10 | 11 | 0.1 | 0.1 | 0.9 | 99 | 52.4 | 50 |
| 70 | 211 | bwa | 6 | 0.1 | 12 | 4 | 99.2 | 60 | 1.6 | 25 | 10 | 12 | 0.1 | 0.1 | 0.8 | 99 | 54.5 | 50 |
| 70 | 212 | bowtie2 | 6 | 0.1 | 9 | 4 | 98.9 | 60 | 1.6 | 30.8 | 10 | 9 | 0.1 | 0.1 | 1.1 | 99 | 47.4 | 50 |
| 70 | 212 | bwa | 6 | 0.1 | 11 | 4 | 99.1 | 60 | 1.6 | 26.7 | 10 | 11 | 0.1 | 0.1 | 0.9 | 99 | 52.4 | 50 |
| 70 | 213 | bowtie2 | 6 | 0.1 | 11 | 4 | 99.1 | 60 | 1.6 | 26.7 | 10 | 11 | 0.1 | 0.1 | 0.9 | 99 | 52.4 | 50 |
| 70 | 213 | bwa | 6 | 0.1 | 13 | 4 | 99.2 | 60 | 1.6 | 23.5 | 10 | 13 | 0.1 | 0.1 | 0.8 | 99 | 56.5 | 50 |
| 70 | 214 | bowtie2 | 6 | 0.1 | 9 | 4 | 98.9 | 60 | 1.6 | 30.8 | 10 | 9 | 0.1 | 0.1 | 1.1 | 99 | 47.4 | 50 |
| 70 | 214 | bwa | 6 | 0.1 | 12 | 4 | 99.2 | 60 | 1.6 | 25 | 10 | 12 | 0.1 | 0.1 | 0.8 | 99 | 54.5 | 50 |
| 70 | 215 | bowtie2 | 6 | 0.1 | 13 | 4 | 99.2 | 60 | 1.6 | 23.5 | 10 | 13 | 0.1 | 0.1 | 0.8 | 99 | 56.5 | 50 |
| 70 | 215 | bwa | 6 | 0.1 | 14 | 4 | 99.3 | 60 | 1.6 | 22.2 | 10 | 14 | 0.1 | 0.1 | 0.7 | 99 | 58.3 | 50 |
| 70 | 216 | bowtie2 | 6 | 0.1 | 15 | 4 | 99.3 | 60 | 1.6 | 21.1 | 10 | 15 | 0.1 | 0.1 | 0.7 | 99 | 60 | 50 |
| 70 | 216 | bwa | 6 | 0.1 | 15 | 4 | 99.3 | 60 | 1.6 | 21.1 | 10 | 15 | 0.1 | 0.1 | 0.7 | 99 | 60 | 50 |
| 70 | 217 | bowtie2 | 6 | 0.1 | 13 | 4 | 99.2 | 60 | 1.6 | 23.5 | 10 | 13 | 0.1 | 0.1 | 0.8 | 99 | 56.5 | 50 |
| 70 | 217 | bwa | 6 | 0.1 | 14 | 4 | 99.3 | 60 | 1.6 | 22.2 | 10 | 14 | 0.1 | 0.1 | 0.7 | 99 | 58.3 | 50 |
| 70 | 218 | bowtie2 | 6 | 0.1 | 10 | 4 | 99 | 60 | 1.6 | 28.6 | 10 | 10 | 0.1 | 0.1 | 1 | 99 | 50 | 50 |
| 70 | 218 | bwa | 6 | 0.1 | 13 | 4 | 99.2 | 60 | 1.6 | 23.5 | 10 | 13 | 0.1 | 0.1 | 0.8 | 99 | 56.5 | 50 |
| 70 | 219 | bowtie2 | 6 | 0.1 | 12 | 4 | 99.2 | 60 | 1.6 | 25 | 10 | 12 | 0.1 | 0.1 | 0.8 | 99 | 54.5 | 50 |
| 70 | 219 | bwa | 6 | 0.1 | 14 | 4 | 99.3 | 60 | 1.6 | 22.2 | 10 | 14 | 0.1 | 0.1 | 0.7 | 99 | 58.3 | 50 |
| 70 | 220 | bowtie2 | 6 | 0.1 | 9 | 4 | 98.9 | 60 | 1.6 | 30.8 | 10 | 9 | 0.1 | 0.1 | 1.1 | 99 | 47.4 | 50 |
| 70 | 220 | bwa | 6 | 0.1 | 11 | 4 | 99.1 | 60 | 1.6 | 26.7 | 10 | 11 | 0.1 | 0.1 | 0.9 | 99 | 52.4 | 50 |
| 70 | 221 | bowtie2 | 6 | 0.1 | 14 | 4 | 99.3 | 60 | 1.6 | 22.2 | 10 | 14 | 0.1 | 0.1 | 0.7 | 99 | 58.3 | 50 |
| 70 | 221 | bwa | 6 | 0.1 | 16 | 4 | 99.4 | 60 | 1.6 | 20 | 10 | 16 | 0.1 | 0.1 | 0.6 | 99 | 61.5 | 50 |
| 70 | 222 | bowtie2 | 6 | 0.1 | 9 | 4 | 98.9 | 60 | 1.6 | 30.8 | 10 | 9 | 0.1 | 0.1 | 1.1 | 99 | 47.4 | 50 |
| 70 | 222 | bwa | 6 | 0.1 | 14 | 4 | 99.3 | 60 | 1.6 | 22.2 | 10 | 14 | 0.1 | 0.1 | 0.7 | 99 | 58.3 | 50 |
| 100 | 202 | bowtie2 | 9 | 0.1 | 12 | 1 | 99.2 | 90 | 1.1 | 7.7 | 10 | 12 | 0.1 | 0.1 | 0.8 | 99 | 54.5 | 50 |
| 100 | 202 | bwa | 9 | 0.1 | 16 | 1 | 99.4 | 90 | 1.1 | 5.9 | 10 | 16 | 0.1 | 0.1 | 0.6 | 99 | 61.5 | 50 |
| 100 | 203 | bowtie2 | 9 | 0.1 | 13 | 1 | 99.2 | 90 | 1.1 | 7.1 | 10 | 13 | 0.1 | 0.1 | 0.8 | 99 | 56.5 | 50 |
| 100 | 203 | bwa | 9 | 0.1 | 14 | 1 | 99.3 | 90 | 1.1 | 6.7 | 10 | 14 | 0.1 | 0.1 | 0.7 | 99 | 58.3 | 50 |
| 100 | 204 | bowtie2 | 9 | 0.1 | 14 | 1 | 99.3 | 90 | 1.1 | 6.7 | 10 | 14 | 0.1 | 0.1 | 0.7 | 99 | 58.3 | 50 |
| 100 | 204 | bwa | 10 | 0.1 | 15 | 0.1 | 99.3 | 99 | 1 | 0.7 | 10 | 15 | 0.1 | 0.1 | 0.7 | 99 | 60 | 50 |
| 100 | 205 | bowtie2 | 8 | 0.1 | 14 | 2 | 99.3 | 80 | 1.2 | 12.5 | 10 | 14 | 0.1 | 0.1 | 0.7 | 99 | 58.3 | 50 |
| 100 | 205 | bwa | 9 | 0.1 | 13 | 1 | 99.2 | 90 | 1.1 | 7.1 | 10 | 13 | 0.1 | 0.1 | 0.8 | 99 | 56.5 | 50 |
| 100 | 206 | bowtie2 | 8 | 0.1 | 18 | 2 | 99.4 | 80 | 1.2 | 10 | 10 | 18 | 0.1 | 0.1 | 0.6 | 99 | 64.3 | 50 |
| 100 | 206 | bwa | 9 | 0.1 | 14 | 1 | 99.3 | 90 | 1.1 | 6.7 | 10 | 14 | 0.1 | 0.1 | 0.7 | 99 | 58.3 | 50 |
| 100 | 207 | bowtie2 | 8 | 0.1 | 12 | 2 | 99.2 | 80 | 1.2 | 14.3 | 10 | 12 | 0.1 | 0.1 | 0.8 | 99 | 54.5 | 50 |
| 100 | 207 | bwa | 9 | 0.1 | 15 | 1 | 99.3 | 90 | 1.1 | 6.3 | 10 | 15 | 0.1 | 0.1 | 0.7 | 99 | 60 | 50 |
| 100 | 208 | bowtie2 | 9 | 0.1 | 11 | 1 | 99.1 | 90 | 1.1 | 8.3 | 10 | 11 | 0.1 | 0.1 | 0.9 | 99 | 52.4 | 50 |
| 100 | 208 | bwa | 9 | 0.1 | 11 | 1 | 99.1 | 90 | 1.1 | 8.3 | 10 | 11 | 0.1 | 0.1 | 0.9 | 99 | 52.4 | 50 |
| 100 | 209 | bowtie2 | 10 | 0.1 | 13 | 0.1 | 99.2 | 99 | 1 | 0.8 | 10 | 13 | 0.1 | 0.1 | 0.8 | 99 | 56.5 | 50 |
| 100 | 209 | bwa | 9 | 0.1 | 14 | 1 | 99.3 | 90 | 1.1 | 6.7 | 10 | 14 | 0.1 | 0.1 | 0.7 | 99 | 58.3 | 50 |
| 100 | 210 | bowtie2 | 8 | 0.1 | 13 | 2 | 99.2 | 80 | 1.2 | 13.3 | 10 | 13 | 0.1 | 0.1 | 0.8 | 99 | 56.5 | 50 |
| 100 | 210 | bwa | 9 | 0.1 | 16 | 1 | 99.4 | 90 | 1.1 | 5.9 | 10 | 16 | 0.1 | 0.1 | 0.6 | 99 | 61.5 | 50 |
| 100 | 211 | bowtie2 | 9 | 0.1 | 12 | 1 | 99.2 | 90 | 1.1 | 7.7 | 10 | 12 | 0.1 | 0.1 | 0.8 | 99 | 54.5 | 50 |
| 100 | 211 | bwa | 10 | 0.1 | 12 | 0.1 | 99.2 | 99 | 1 | 0.8 | 10 | 12 | 0.1 | 0.1 | 0.8 | 99 | 54.5 | 50 |
| 100 | 212 | bowtie2 | 9 | 0.1 | 11 | 1 | 99.1 | 90 | 1.1 | 8.3 | 10 | 11 | 0.1 | 0.1 | 0.9 | 99 | 52.4 | 50 |
| 100 | 212 | bwa | 9 | 0.1 | 15 | 1 | 99.3 | 90 | 1.1 | 6.3 | 10 | 15 | 0.1 | 0.1 | 0.7 | 99 | 60 | 50 |
| 100 | 213 | bowtie2 | 9 | 0.1 | 12 | 1 | 99.2 | 90 | 1.1 | 7.7 | 10 | 12 | 0.1 | 0.1 | 0.8 | 99 | 54.5 | 50 |
| 100 | 213 | bwa | 8 | 0.1 | 17 | 2 | 99.4 | 80 | 1.2 | 10.5 | 10 | 17 | 0.1 | 0.1 | 0.6 | 99 | 63 | 50 |
| 100 | 214 | bowtie2 | 9 | 0.1 | 11 | 1 | 99.1 | 90 | 1.1 | 8.3 | 10 | 11 | 0.1 | 0.1 | 0.9 | 99 | 52.4 | 50 |
| 100 | 214 | bwa | 9 | 0.1 | 18 | 1 | 99.4 | 90 | 1.1 | 5.3 | 10 | 18 | 0.1 | 0.1 | 0.6 | 99 | 64.3 | 50 |
| 100 | 215 | bowtie2 | 9 | 0.1 | 14 | 1 | 99.3 | 90 | 1.1 | 6.7 | 10 | 14 | 0.1 | 0.1 | 0.7 | 99 | 58.3 | 50 |
| 100 | 215 | bwa | 9 | 0.1 | 15 | 1 | 99.3 | 90 | 1.1 | 6.3 | 10 | 15 | 0.1 | 0.1 | 0.7 | 99 | 60 | 50 |
| 100 | 216 | bowtie2 | 9 | 0.1 | 14 | 1 | 99.3 | 90 | 1.1 | 6.7 | 10 | 14 | 0.1 | 0.1 | 0.7 | 99 | 58.3 | 50 |
| 100 | 216 | bwa | 9 | 0.1 | 17 | 1 | 99.4 | 90 | 1.1 | 5.6 | 10 | 17 | 0.1 | 0.1 | 0.6 | 99 | 63 | 50 |
| 100 | 217 | bowtie2 | 9 | 0.1 | 15 | 1 | 99.3 | 90 | 1.1 | 6.3 | 10 | 15 | 0.1 | 0.1 | 0.7 | 99 | 60 | 50 |
| 100 | 217 | bwa | 9 | 0.1 | 13 | 1 | 99.2 | 90 | 1.1 | 7.1 | 10 | 13 | 0.1 | 0.1 | 0.8 | 99 | 56.5 | 50 |
| 100 | 218 | bowtie2 | 8 | 0.1 | 13 | 2 | 99.2 | 80 | 1.2 | 13.3 | 10 | 13 | 0.1 | 0.1 | 0.8 | 99 | 56.5 | 50 |
| 100 | 218 | bwa | 9 | 0.1 | 14 | 1 | 99.3 | 90 | 1.1 | 6.7 | 10 | 14 | 0.1 | 0.1 | 0.7 | 99 | 58.3 | 50 |
| 100 | 219 | bowtie2 | 9 | 0.1 | 14 | 1 | 99.3 | 90 | 1.1 | 6.7 | 10 | 14 | 0.1 | 0.1 | 0.7 | 99 | 58.3 | 50 |
| 100 | 219 | bwa | 10 | 0.1 | 12 | 0.1 | 99.2 | 99 | 1 | 0.8 | 10 | 12 | 0.1 | 0.1 | 0.8 | 99 | 54.5 | 50 |
| 100 | 220 | bowtie2 | 9 | 0.1 | 10 | 1 | 99 | 90 | 1.1 | 9.1 | 10 | 10 | 0.1 | 0.1 | 1 | 99 | 50 | 50 |
| 100 | 220 | bwa | 9 | 0.1 | 16 | 1 | 99.4 | 90 | 1.1 | 5.9 | 10 | 16 | 0.1 | 0.1 | 0.6 | 99 | 61.5 | 50 |
| 100 | 221 | bowtie2 | 9 | 0.1 | 15 | 1 | 99.3 | 90 | 1.1 | 6.3 | 10 | 15 | 0.1 | 0.1 | 0.7 | 99 | 60 | 50 |
| 100 | 221 | bwa | 9 | 0.1 | 14 | 1 | 99.3 | 90 | 1.1 | 6.7 | 10 | 14 | 0.1 | 0.1 | 0.7 | 99 | 58.3 | 50 |
| 100 | 222 | bowtie2 | 10 | 0.1 | 12 | 0.1 | 99.2 | 99 | 1 | 0.8 | 10 | 12 | 0.1 | 0.1 | 0.8 | 99 | 54.5 | 50 |
| 100 | 222 | bwa | 10 | 0.1 | 18 | 0.1 | 99.4 | 99 | 1 | 0.6 | 10 | 18 | 0.1 | 0.1 | 0.6 | 99 | 64.3 | 50 |
| 200 | 202 | bowtie2 | 10 | 0.1 | 13 | 0.1 | 99.2 | 99 | 1 | 0.8 | 10 | 13 | 0.1 | 0.1 | 0.8 | 99 | 56.5 | 50 |
| 200 | 202 | bwa | 10 | 0.1 | 15 | 0.1 | 99.3 | 99 | 1 | 0.7 | 10 | 15 | 0.1 | 0.1 | 0.7 | 99 | 60 | 50 |
| 200 | 203 | bowtie2 | 10 | 0.1 | 15 | 0.1 | 99.3 | 99 | 1 | 0.7 | 10 | 15 | 0.1 | 0.1 | 0.7 | 99 | 60 | 50 |
| 200 | 203 | bwa | 10 | 0.1 | 16 | 0.1 | 99.4 | 99 | 1 | 0.6 | 10 | 16 | 0.1 | 0.1 | 0.6 | 99 | 61.5 | 50 |
| 200 | 204 | bowtie2 | 10 | 0.1 | 15 | 0.1 | 99.3 | 99 | 1 | 0.7 | 10 | 15 | 0.1 | 0.1 | 0.7 | 99 | 60 | 50 |
| 200 | 204 | bwa | 10 | 0.1 | 17 | 0.1 | 99.4 | 99 | 1 | 0.6 | 10 | 17 | 0.1 | 0.1 | 0.6 | 99 | 63 | 50 |
| 200 | 205 | bowtie2 | 10 | 0.1 | 16 | 0.1 | 99.4 | 99 | 1 | 0.6 | 10 | 16 | 0.1 | 0.1 | 0.6 | 99 | 61.5 | 50 |
| 200 | 205 | bwa | 10 | 0.1 | 16 | 0.1 | 99.4 | 99 | 1 | 0.6 | 10 | 16 | 0.1 | 0.1 | 0.6 | 99 | 61.5 | 50 |
| 200 | 206 | bowtie2 | 10 | 0.1 | 18 | 0.1 | 99.4 | 99 | 1 | 0.6 | 10 | 18 | 0.1 | 0.1 | 0.6 | 99 | 64.3 | 50 |
| 200 | 206 | bwa | 10 | 0.1 | 18 | 0.1 | 99.4 | 99 | 1 | 0.6 | 10 | 18 | 0.1 | 0.1 | 0.6 | 99 | 64.3 | 50 |
| 200 | 207 | bowtie2 | 10 | 0.1 | 13 | 0.1 | 99.2 | 99 | 1 | 0.8 | 10 | 13 | 0.1 | 0.1 | 0.8 | 99 | 56.5 | 50 |
| 200 | 207 | bwa | 10 | 0.1 | 12 | 0.1 | 99.2 | 99 | 1 | 0.8 | 10 | 12 | 0.1 | 0.1 | 0.8 | 99 | 54.5 | 50 |
| 200 | 208 | bowtie2 | 10 | 0.1 | 12 | 0.1 | 99.2 | 99 | 1 | 0.8 | 10 | 12 | 0.1 | 0.1 | 0.8 | 99 | 54.5 | 50 |
| 200 | 208 | bwa | 10 | 0.1 | 14 | 0.1 | 99.3 | 99 | 1 | 0.7 | 10 | 14 | 0.1 | 0.1 | 0.7 | 99 | 58.3 | 50 |
| 200 | 209 | bowtie2 | 10 | 0.1 | 15 | 0.1 | 99.3 | 99 | 1 | 0.7 | 10 | 15 | 0.1 | 0.1 | 0.7 | 99 | 60 | 50 |
| 200 | 209 | bwa | 10 | 0.1 | 18 | 0.1 | 99.4 | 99 | 1 | 0.6 | 10 | 18 | 0.1 | 0.1 | 0.6 | 99 | 64.3 | 50 |
| 200 | 210 | bowtie2 | 10 | 0.1 | 16 | 0.1 | 99.4 | 99 | 1 | 0.6 | 10 | 16 | 0.1 | 0.1 | 0.6 | 99 | 61.5 | 50 |
| 200 | 210 | bwa | 10 | 0.1 | 17 | 0.1 | 99.4 | 99 | 1 | 0.6 | 10 | 17 | 0.1 | 0.1 | 0.6 | 99 | 63 | 50 |
| 200 | 211 | bowtie2 | 10 | 0.1 | 13 | 0.1 | 99.2 | 99 | 1 | 0.8 | 10 | 13 | 0.1 | 0.1 | 0.8 | 99 | 56.5 | 50 |
| 200 | 211 | bwa | 10 | 0.1 | 16 | 0.1 | 99.4 | 99 | 1 | 0.6 | 10 | 16 | 0.1 | 0.1 | 0.6 | 99 | 61.5 | 50 |
| 200 | 212 | bowtie2 | 10 | 0.1 | 13 | 0.1 | 99.2 | 99 | 1 | 0.8 | 10 | 13 | 0.1 | 0.1 | 0.8 | 99 | 56.5 | 50 |
| 200 | 212 | bwa | 10 | 0.1 | 17 | 0.1 | 99.4 | 99 | 1 | 0.6 | 10 | 17 | 0.1 | 0.1 | 0.6 | 99 | 63 | 50 |
| 200 | 213 | bowtie2 | 10 | 0.1 | 13 | 0.1 | 99.2 | 99 | 1 | 0.8 | 10 | 13 | 0.1 | 0.1 | 0.8 | 99 | 56.5 | 50 |
| 200 | 213 | bwa | 10 | 0.1 | 15 | 0.1 | 99.3 | 99 | 1 | 0.7 | 10 | 15 | 0.1 | 0.1 | 0.7 | 99 | 60 | 50 |
| 200 | 214 | bowtie2 | 10 | 0.1 | 12 | 0.1 | 99.2 | 99 | 1 | 0.8 | 10 | 12 | 0.1 | 0.1 | 0.8 | 99 | 54.5 | 50 |
| 200 | 214 | bwa | 10 | 0.1 | 16 | 0.1 | 99.4 | 99 | 1 | 0.6 | 10 | 16 | 0.1 | 0.1 | 0.6 | 99 | 61.5 | 50 |
| 200 | 215 | bowtie2 | 10 | 0.1 | 15 | 0.1 | 99.3 | 99 | 1 | 0.7 | 10 | 15 | 0.1 | 0.1 | 0.7 | 99 | 60 | 50 |
| 200 | 215 | bwa | 10 | 0.1 | 18 | 0.1 | 99.4 | 99 | 1 | 0.6 | 10 | 18 | 0.1 | 0.1 | 0.6 | 99 | 64.3 | 50 |
| 200 | 216 | bowtie2 | 10 | 0.1 | 15 | 0.1 | 99.3 | 99 | 1 | 0.7 | 10 | 15 | 0.1 | 0.1 | 0.7 | 99 | 60 | 50 |
| 200 | 216 | bwa | 10 | 0.1 | 20 | 0.1 | 99.5 | 99 | 1 | 0.5 | 10 | 20 | 0.1 | 0.1 | 0.5 | 99 | 66.7 | 50 |
| 200 | 217 | bowtie2 | 10 | 0.1 | 15 | 0.1 | 99.3 | 99 | 1 | 0.7 | 10 | 15 | 0.1 | 0.1 | 0.7 | 99 | 60 | 50 |
| 200 | 217 | bwa | 10 | 0.1 | 16 | 0.1 | 99.4 | 99 | 1 | 0.6 | 10 | 16 | 0.1 | 0.1 | 0.6 | 99 | 61.5 | 50 |
| 200 | 218 | bowtie2 | 10 | 0.1 | 13 | 0.1 | 99.2 | 99 | 1 | 0.8 | 10 | 13 | 0.1 | 0.1 | 0.8 | 99 | 56.5 | 50 |
| 200 | 218 | bwa | 10 | 0.1 | 17 | 0.1 | 99.4 | 99 | 1 | 0.6 | 10 | 17 | 0.1 | 0.1 | 0.6 | 99 | 63 | 50 |
| 200 | 219 | bowtie2 | 10 | 0.1 | 15 | 0.1 | 99.3 | 99 | 1 | 0.7 | 10 | 15 | 0.1 | 0.1 | 0.7 | 99 | 60 | 50 |
| 200 | 219 | bwa | 10 | 0.1 | 17 | 0.1 | 99.4 | 99 | 1 | 0.6 | 10 | 17 | 0.1 | 0.1 | 0.6 | 99 | 63 | 50 |
| 200 | 220 | bowtie2 | 10 | 0.1 | 11 | 0.1 | 99.1 | 99 | 1 | 0.9 | 10 | 11 | 0.1 | 0.1 | 0.9 | 99 | 52.4 | 50 |
| 200 | 220 | bwa | 10 | 0.1 | 18 | 0.1 | 99.4 | 99 | 1 | 0.6 | 10 | 18 | 0.1 | 0.1 | 0.6 | 99 | 64.3 | 50 |
| 200 | 221 | bowtie2 | 10 | 0.1 | 16 | 0.1 | 99.4 | 99 | 1 | 0.6 | 10 | 16 | 0.1 | 0.1 | 0.6 | 99 | 61.5 | 50 |
| 200 | 221 | bwa | 10 | 0.1 | 16 | 0.1 | 99.4 | 99 | 1 | 0.6 | 10 | 16 | 0.1 | 0.1 | 0.6 | 99 | 61.5 | 50 |
| 200 | 222 | bowtie2 | 10 | 0.1 | 12 | 0.1 | 99.2 | 99 | 1 | 0.8 | 10 | 12 | 0.1 | 0.1 | 0.8 | 99 | 54.5 | 50 |
| 200 | 222 | bwa | 10 | 0.1 | 17 | 0.1 | 99.4 | 99 | 1 | 0.6 | 10 | 17 | 0.1 | 0.1 | 0.6 | 99 | 63 | 50 |
| 300 | 202 | bowtie2 | 10 | 0.1 | 15 | 0.1 | 99.3 | 99 | 1 | 0.7 | 10 | 15 | 0.1 | 0.1 | 0.7 | 99 | 60 | 50 |
| 300 | 202 | bwa | 10 | 0.1 | 18 | 0.1 | 99.4 | 99 | 1 | 0.6 | 10 | 18 | 0.1 | 0.1 | 0.6 | 99 | 64.3 | 50 |
| 300 | 203 | bowtie2 | 10 | 0.1 | 17 | 0.1 | 99.4 | 99 | 1 | 0.6 | 10 | 17 | 0.1 | 0.1 | 0.6 | 99 | 63 | 50 |
| 300 | 203 | bwa | 10 | 0.1 | 15 | 0.1 | 99.3 | 99 | 1 | 0.7 | 10 | 15 | 0.1 | 0.1 | 0.7 | 99 | 60 | 50 |
| 300 | 204 | bowtie2 | 10 | 0.1 | 15 | 0.1 | 99.3 | 99 | 1 | 0.7 | 10 | 15 | 0.1 | 0.1 | 0.7 | 99 | 60 | 50 |
| 300 | 204 | bwa | 10 | 0.1 | 18 | 0.1 | 99.4 | 99 | 1 | 0.6 | 10 | 18 | 0.1 | 0.1 | 0.6 | 99 | 64.3 | 50 |
| 300 | 205 | bowtie2 | 10 | 0.1 | 15 | 0.1 | 99.3 | 99 | 1 | 0.7 | 10 | 15 | 0.1 | 0.1 | 0.7 | 99 | 60 | 50 |
| 300 | 205 | bwa | 10 | 0.1 | 19 | 0.1 | 99.5 | 99 | 1 | 0.5 | 10 | 19 | 0.1 | 0.1 | 0.5 | 99 | 65.5 | 50 |
| 300 | 206 | bowtie2 | 10 | 0.1 | 19 | 0.1 | 99.5 | 99 | 1 | 0.5 | 10 | 19 | 0.1 | 0.1 | 0.5 | 99 | 65.5 | 50 |
| 300 | 206 | bwa | 10 | 0.1 | 16 | 0.1 | 99.4 | 99 | 1 | 0.6 | 10 | 16 | 0.1 | 0.1 | 0.6 | 99 | 61.5 | 50 |
| 300 | 207 | bowtie2 | 10 | 0.1 | 13 | 0.1 | 99.2 | 99 | 1 | 0.8 | 10 | 13 | 0.1 | 0.1 | 0.8 | 99 | 56.5 | 50 |
| 300 | 207 | bwa | 10 | 0.1 | 15 | 0.1 | 99.3 | 99 | 1 | 0.7 | 10 | 15 | 0.1 | 0.1 | 0.7 | 99 | 60 | 50 |
| 300 | 208 | bowtie2 | 10 | 0.1 | 12 | 0.1 | 99.2 | 99 | 1 | 0.8 | 10 | 12 | 0.1 | 0.1 | 0.8 | 99 | 54.5 | 50 |
| 300 | 208 | bwa | 10 | 0.1 | 15 | 0.1 | 99.3 | 99 | 1 | 0.7 | 10 | 15 | 0.1 | 0.1 | 0.7 | 99 | 60 | 50 |
| 300 | 209 | bowtie2 | 10 | 0.1 | 15 | 0.1 | 99.3 | 99 | 1 | 0.7 | 10 | 15 | 0.1 | 0.1 | 0.7 | 99 | 60 | 50 |
| 300 | 209 | bwa | 10 | 0.1 | 17 | 0.1 | 99.4 | 99 | 1 | 0.6 | 10 | 17 | 0.1 | 0.1 | 0.6 | 99 | 63 | 50 |
| 300 | 210 | bowtie2 | 10 | 0.1 | 16 | 0.1 | 99.4 | 99 | 1 | 0.6 | 10 | 16 | 0.1 | 0.1 | 0.6 | 99 | 61.5 | 50 |
| 300 | 210 | bwa | 10 | 0.1 | 19 | 0.1 | 99.5 | 99 | 1 | 0.5 | 10 | 19 | 0.1 | 0.1 | 0.5 | 99 | 65.5 | 50 |
| 300 | 211 | bowtie2 | 10 | 0.1 | 13 | 0.1 | 99.2 | 99 | 1 | 0.8 | 10 | 13 | 0.1 | 0.1 | 0.8 | 99 | 56.5 | 50 |
| 300 | 211 | bwa | 10 | 0.1 | 14 | 0.1 | 99.3 | 99 | 1 | 0.7 | 10 | 14 | 0.1 | 0.1 | 0.7 | 99 | 58.3 | 50 |
| 300 | 212 | bowtie2 | 10 | 0.1 | 15 | 0.1 | 99.3 | 99 | 1 | 0.7 | 10 | 15 | 0.1 | 0.1 | 0.7 | 99 | 60 | 50 |
| 300 | 212 | bwa | 10 | 0.1 | 13 | 0.1 | 99.2 | 99 | 1 | 0.8 | 10 | 13 | 0.1 | 0.1 | 0.8 | 99 | 56.5 | 50 |
| 300 | 213 | bowtie2 | 10 | 0.1 | 14 | 0.1 | 99.3 | 99 | 1 | 0.7 | 10 | 14 | 0.1 | 0.1 | 0.7 | 99 | 58.3 | 50 |
| 300 | 213 | bwa | 10 | 0.1 | 16 | 0.1 | 99.4 | 99 | 1 | 0.6 | 10 | 16 | 0.1 | 0.1 | 0.6 | 99 | 61.5 | 50 |
| 300 | 214 | bowtie2 | 10 | 0.1 | 13 | 0.1 | 99.2 | 99 | 1 | 0.8 | 10 | 13 | 0.1 | 0.1 | 0.8 | 99 | 56.5 | 50 |
| 300 | 214 | bwa | 10 | 0.1 | 18 | 0.1 | 99.4 | 99 | 1 | 0.6 | 10 | 18 | 0.1 | 0.1 | 0.6 | 99 | 64.3 | 50 |
| 300 | 215 | bowtie2 | 10 | 0.1 | 16 | 0.1 | 99.4 | 99 | 1 | 0.6 | 10 | 16 | 0.1 | 0.1 | 0.6 | 99 | 61.5 | 50 |
| 300 | 215 | bwa | 10 | 0.1 | 22 | 0.1 | 99.5 | 99 | 1 | 0.5 | 10 | 22 | 0.1 | 0.1 | 0.5 | 99 | 68.8 | 50 |
| 300 | 216 | bowtie2 | 10 | 0.1 | 16 | 0.1 | 99.4 | 99 | 1 | 0.6 | 10 | 16 | 0.1 | 0.1 | 0.6 | 99 | 61.5 | 50 |
| 300 | 216 | bwa | 10 | 0.1 | 16 | 0.1 | 99.4 | 99 | 1 | 0.6 | 10 | 16 | 0.1 | 0.1 | 0.6 | 99 | 61.5 | 50 |
| 300 | 217 | bowtie2 | 10 | 0.1 | 15 | 0.1 | 99.3 | 99 | 1 | 0.7 | 10 | 15 | 0.1 | 0.1 | 0.7 | 99 | 60 | 50 |
| 300 | 217 | bwa | 10 | 0.1 | 17 | 0.1 | 99.4 | 99 | 1 | 0.6 | 10 | 17 | 0.1 | 0.1 | 0.6 | 99 | 63 | 50 |
| 300 | 218 | bowtie2 | 10 | 0.1 | 13 | 0.1 | 99.2 | 99 | 1 | 0.8 | 10 | 13 | 0.1 | 0.1 | 0.8 | 99 | 56.5 | 50 |
| 300 | 218 | bwa | 10 | 0.1 | 13 | 0.1 | 99.2 | 99 | 1 | 0.8 | 10 | 13 | 0.1 | 0.1 | 0.8 | 99 | 56.5 | 50 |
| 300 | 219 | bowtie2 | 10 | 0.1 | 16 | 0.1 | 99.4 | 99 | 1 | 0.6 | 10 | 16 | 0.1 | 0.1 | 0.6 | 99 | 61.5 | 50 |
| 300 | 219 | bwa | 10 | 0.1 | 16 | 0.1 | 99.4 | 99 | 1 | 0.6 | 10 | 16 | 0.1 | 0.1 | 0.6 | 99 | 61.5 | 50 |
| 300 | 220 | bowtie2 | 10 | 0.1 | 12 | 0.1 | 99.2 | 99 | 1 | 0.8 | 10 | 12 | 0.1 | 0.1 | 0.8 | 99 | 54.5 | 50 |
| 300 | 220 | bwa | 10 | 0.1 | 15 | 0.1 | 99.3 | 99 | 1 | 0.7 | 10 | 15 | 0.1 | 0.1 | 0.7 | 99 | 60 | 50 |
| 300 | 221 | bowtie2 | 10 | 0.1 | 17 | 0.1 | 99.4 | 99 | 1 | 0.6 | 10 | 17 | 0.1 | 0.1 | 0.6 | 99 | 63 | 50 |
| 300 | 221 | bwa | 10 | 0.1 | 14 | 0.1 | 99.3 | 99 | 1 | 0.7 | 10 | 14 | 0.1 | 0.1 | 0.7 | 99 | 58.3 | 50 |
| 300 | 222 | bowtie2 | 10 | 0.1 | 14 | 0.1 | 99.3 | 99 | 1 | 0.7 | 10 | 14 | 0.1 | 0.1 | 0.7 | 99 | 58.3 | 50 |
| 300 | 222 | bwa | 10 | 0.1 | 17 | 0.1 | 99.4 | 99 | 1 | 0.6 | 10 | 17 | 0.1 | 0.1 | 0.6 | 99 | 63 | 50 |
| 400 | 202 | bowtie2 | 10 | 0.1 | 15 | 0.1 | 99.3 | 99 | 1 | 0.7 | 10 | 15 | 0.1 | 0.1 | 0.7 | 99 | 60 | 50 |
| 400 | 202 | bwa | 10 | 0.1 | 18 | 0.1 | 99.4 | 99 | 1 | 0.6 | 10 | 18 | 0.1 | 0.1 | 0.6 | 99 | 64.3 | 50 |
| 400 | 203 | bowtie2 | 10 | 0.1 | 17 | 0.1 | 99.4 | 99 | 1 | 0.6 | 10 | 17 | 0.1 | 0.1 | 0.6 | 99 | 63 | 50 |
| 400 | 203 | bwa | 10 | 0.1 | 19 | 0.1 | 99.5 | 99 | 1 | 0.5 | 10 | 19 | 0.1 | 0.1 | 0.5 | 99 | 65.5 | 50 |
| 400 | 204 | bowtie2 | 10 | 0.1 | 16 | 0.1 | 99.4 | 99 | 1 | 0.6 | 10 | 16 | 0.1 | 0.1 | 0.6 | 99 | 61.5 | 50 |
| 400 | 204 | bwa | 10 | 0.1 | 16 | 0.1 | 99.4 | 99 | 1 | 0.6 | 10 | 16 | 0.1 | 0.1 | 0.6 | 99 | 61.5 | 50 |
| 400 | 205 | bowtie2 | 10 | 0.1 | 16 | 0.1 | 99.4 | 99 | 1 | 0.6 | 10 | 16 | 0.1 | 0.1 | 0.6 | 99 | 61.5 | 50 |
| 400 | 205 | bwa | 10 | 0.1 | 16 | 0.1 | 99.4 | 99 | 1 | 0.6 | 10 | 16 | 0.1 | 0.1 | 0.6 | 99 | 61.5 | 50 |
| 400 | 206 | bowtie2 | 10 | 0.1 | 19 | 0.1 | 99.5 | 99 | 1 | 0.5 | 10 | 19 | 0.1 | 0.1 | 0.5 | 99 | 65.5 | 50 |
| 400 | 206 | bwa | 10 | 0.1 | 20 | 0.1 | 99.5 | 99 | 1 | 0.5 | 10 | 20 | 0.1 | 0.1 | 0.5 | 99 | 66.7 | 50 |
| 400 | 207 | bowtie2 | 10 | 0.1 | 15 | 0.1 | 99.3 | 99 | 1 | 0.7 | 10 | 15 | 0.1 | 0.1 | 0.7 | 99 | 60 | 50 |
| 400 | 207 | bwa | 10 | 0.1 | 12 | 0.1 | 99.2 | 99 | 1 | 0.8 | 10 | 12 | 0.1 | 0.1 | 0.8 | 99 | 54.5 | 50 |
| 400 | 208 | bowtie2 | 10 | 0.1 | 16 | 0.1 | 99.4 | 99 | 1 | 0.6 | 10 | 16 | 0.1 | 0.1 | 0.6 | 99 | 61.5 | 50 |
| 400 | 208 | bwa | 10 | 0.1 | 12 | 0.1 | 99.2 | 99 | 1 | 0.8 | 10 | 12 | 0.1 | 0.1 | 0.8 | 99 | 54.5 | 50 |
| 400 | 209 | bowtie2 | 10 | 0.1 | 16 | 0.1 | 99.4 | 99 | 1 | 0.6 | 10 | 16 | 0.1 | 0.1 | 0.6 | 99 | 61.5 | 50 |
| 400 | 209 | bwa | 10 | 0.1 | 18 | 0.1 | 99.4 | 99 | 1 | 0.6 | 10 | 18 | 0.1 | 0.1 | 0.6 | 99 | 64.3 | 50 |
| 400 | 210 | bowtie2 | 10 | 0.1 | 17 | 0.1 | 99.4 | 99 | 1 | 0.6 | 10 | 17 | 0.1 | 0.1 | 0.6 | 99 | 63 | 50 |
| 400 | 210 | bwa | 10 | 0.1 | 18 | 0.1 | 99.4 | 99 | 1 | 0.6 | 10 | 18 | 0.1 | 0.1 | 0.6 | 99 | 64.3 | 50 |
| 400 | 211 | bowtie2 | 10 | 0.1 | 13 | 0.1 | 99.2 | 99 | 1 | 0.8 | 10 | 13 | 0.1 | 0.1 | 0.8 | 99 | 56.5 | 50 |
| 400 | 211 | bwa | 10 | 0.1 | 15 | 0.1 | 99.3 | 99 | 1 | 0.7 | 10 | 15 | 0.1 | 0.1 | 0.7 | 99 | 60 | 50 |
| 400 | 212 | bowtie2 | 10 | 0.1 | 13 | 0.1 | 99.2 | 99 | 1 | 0.8 | 10 | 13 | 0.1 | 0.1 | 0.8 | 99 | 56.5 | 50 |
| 400 | 212 | bwa | 10 | 0.1 | 15 | 0.1 | 99.3 | 99 | 1 | 0.7 | 10 | 15 | 0.1 | 0.1 | 0.7 | 99 | 60 | 50 |
| 400 | 213 | bowtie2 | 10 | 0.1 | 18 | 0.1 | 99.4 | 99 | 1 | 0.6 | 10 | 18 | 0.1 | 0.1 | 0.6 | 99 | 64.3 | 50 |
| 400 | 213 | bwa | 10 | 0.1 | 17 | 0.1 | 99.4 | 99 | 1 | 0.6 | 10 | 17 | 0.1 | 0.1 | 0.6 | 99 | 63 | 50 |
| 400 | 214 | bowtie2 | 10 | 0.1 | 13 | 0.1 | 99.2 | 99 | 1 | 0.8 | 10 | 13 | 0.1 | 0.1 | 0.8 | 99 | 56.5 | 50 |
| 400 | 214 | bwa | 10 | 0.1 | 21 | 0.1 | 99.5 | 99 | 1 | 0.5 | 10 | 21 | 0.1 | 0.1 | 0.5 | 99 | 67.7 | 50 |
| 400 | 215 | bowtie2 | 10 | 0.1 | 19 | 0.1 | 99.5 | 99 | 1 | 0.5 | 10 | 19 | 0.1 | 0.1 | 0.5 | 99 | 65.5 | 50 |
| 400 | 215 | bwa | 10 | 0.1 | 20 | 0.1 | 99.5 | 99 | 1 | 0.5 | 10 | 20 | 0.1 | 0.1 | 0.5 | 99 | 66.7 | 50 |
| 400 | 216 | bowtie2 | 10 | 0.1 | 18 | 0.1 | 99.4 | 99 | 1 | 0.6 | 10 | 18 | 0.1 | 0.1 | 0.6 | 99 | 64.3 | 50 |
| 400 | 216 | bwa | 10 | 1 | 19 | 0.1 | 95 | 99 | 9.1 | 0.5 | 10 | 19 | 0.1 | 0.1 | 0.5 | 99 | 65.5 | 50 |
| 400 | 217 | bowtie2 | 10 | 1 | 15 | 0.1 | 93.8 | 99 | 9.1 | 0.7 | 10 | 15 | 0.1 | 0.1 | 0.7 | 99 | 60 | 50 |
| 400 | 217 | bwa | 10 | 0.1 | 22 | 0.1 | 99.5 | 99 | 1 | 0.5 | 10 | 22 | 0.1 | 0.1 | 0.5 | 99 | 68.8 | 50 |
| 400 | 218 | bowtie2 | 10 | 0.1 | 14 | 0.1 | 99.3 | 99 | 1 | 0.7 | 10 | 14 | 0.1 | 0.1 | 0.7 | 99 | 58.3 | 50 |
| 400 | 218 | bwa | 10 | 0.1 | 18 | 0.1 | 99.4 | 99 | 1 | 0.6 | 10 | 18 | 0.1 | 0.1 | 0.6 | 99 | 64.3 | 50 |
| 400 | 219 | bowtie2 | 10 | 0.1 | 17 | 0.1 | 99.4 | 99 | 1 | 0.6 | 10 | 17 | 0.1 | 0.1 | 0.6 | 99 | 63 | 50 |
| 400 | 219 | bwa | 10 | 0.1 | 16 | 0.1 | 99.4 | 99 | 1 | 0.6 | 10 | 16 | 0.1 | 0.1 | 0.6 | 99 | 61.5 | 50 |
| 400 | 220 | bowtie2 | 10 | 0.1 | 13 | 0.1 | 99.2 | 99 | 1 | 0.8 | 10 | 13 | 0.1 | 0.1 | 0.8 | 99 | 56.5 | 50 |
| 400 | 220 | bwa | 10 | 0.1 | 19 | 0.1 | 99.5 | 99 | 1 | 0.5 | 10 | 19 | 0.1 | 0.1 | 0.5 | 99 | 65.5 | 50 |
| 400 | 221 | bowtie2 | 10 | 0.1 | 19 | 0.1 | 99.5 | 99 | 1 | 0.5 | 10 | 19 | 0.1 | 0.1 | 0.5 | 99 | 65.5 | 50 |
| 400 | 221 | bwa | 10 | 0.1 | 16 | 0.1 | 99.4 | 99 | 1 | 0.6 | 10 | 16 | 0.1 | 0.1 | 0.6 | 99 | 61.5 | 50 |
| 400 | 222 | bowtie2 | 10 | 0.1 | 15 | 0.1 | 99.3 | 99 | 1 | 0.7 | 10 | 15 | 0.1 | 0.1 | 0.7 | 99 | 60 | 50 |
| 400 | 222 | bwa | 10 | 0.1 | 19 | 0.1 | 99.5 | 99 | 1 | 0.5 | 10 | 19 | 0.1 | 0.1 | 0.5 | 99 | 65.5 | 50 |
| 500 | 202 | bowtie2 | 10 | 0.1 | 19 | 0.1 | 99.5 | 99 | 1 | 0.5 | 10 | 19 | 0.1 | 0.1 | 0.5 | 99 | 65.5 | 50 |
| 500 | 202 | bwa | 10 | 1 | 19 | 0.1 | 95 | 99 | 9.1 | 0.5 | 10 | 19 | 0.1 | 0.1 | 0.5 | 99 | 65.5 | 50 |
| 500 | 203 | bowtie2 | 10 | 2 | 19 | 0.1 | 90.5 | 99 | 16.7 | 0.5 | 10 | 19 | 0.1 | 0.1 | 0.5 | 99 | 65.5 | 50 |
| 500 | 203 | bwa | 10 | 1 | 19 | 0.1 | 95 | 99 | 9.1 | 0.5 | 10 | 19 | 0.1 | 0.1 | 0.5 | 99 | 65.5 | 50 |
| 500 | 204 | bowtie2 | 10 | 0.1 | 16 | 0.1 | 99.4 | 99 | 1 | 0.6 | 10 | 16 | 0.1 | 0.1 | 0.6 | 99 | 61.5 | 50 |
| 500 | 204 | bwa | 10 | 1 | 18 | 0.1 | 94.7 | 99 | 9.1 | 0.6 | 10 | 18 | 0.1 | 0.1 | 0.6 | 99 | 64.3 | 50 |
| 500 | 205 | bowtie2 | 10 | 0.1 | 18 | 0.1 | 99.4 | 99 | 1 | 0.6 | 10 | 18 | 0.1 | 0.1 | 0.6 | 99 | 64.3 | 50 |
| 500 | 205 | bwa | 10 | 1 | 20 | 0.1 | 95.2 | 99 | 9.1 | 0.5 | 10 | 20 | 0.1 | 0.1 | 0.5 | 99 | 66.7 | 50 |
| 500 | 206 | bowtie2 | 10 | 0.1 | 21 | 0.1 | 99.5 | 99 | 1 | 0.5 | 10 | 21 | 0.1 | 0.1 | 0.5 | 99 | 67.7 | 50 |
| 500 | 206 | bwa | 10 | 1 | 20 | 0.1 | 95.2 | 99 | 9.1 | 0.5 | 10 | 20 | 0.1 | 0.1 | 0.5 | 99 | 66.7 | 50 |
| 500 | 207 | bowtie2 | 10 | 1 | 14 | 0.1 | 93.3 | 99 | 9.1 | 0.7 | 10 | 14 | 0.1 | 0.1 | 0.7 | 99 | 58.3 | 50 |
| 500 | 207 | bwa | 10 | 1 | 15 | 0.1 | 93.8 | 99 | 9.1 | 0.7 | 10 | 15 | 0.1 | 0.1 | 0.7 | 99 | 60 | 50 |
| 500 | 208 | bowtie2 | 10 | 0.1 | 15 | 0.1 | 99.3 | 99 | 1 | 0.7 | 10 | 15 | 0.1 | 0.1 | 0.7 | 99 | 60 | 50 |
| 500 | 208 | bwa | 10 | 1 | 19 | 0.1 | 95 | 99 | 9.1 | 0.5 | 10 | 19 | 0.1 | 0.1 | 0.5 | 99 | 65.5 | 50 |
| 500 | 209 | bowtie2 | 10 | 0.1 | 17 | 0.1 | 99.4 | 99 | 1 | 0.6 | 10 | 17 | 0.1 | 0.1 | 0.6 | 99 | 63 | 50 |
| 500 | 209 | bwa | 10 | 1 | 21 | 0.1 | 95.5 | 99 | 9.1 | 0.5 | 10 | 21 | 0.1 | 0.1 | 0.5 | 99 | 67.7 | 50 |
| 500 | 210 | bowtie2 | 10 | 1 | 16 | 0.1 | 94.1 | 99 | 9.1 | 0.6 | 10 | 16 | 0.1 | 0.1 | 0.6 | 99 | 61.5 | 50 |
| 500 | 210 | bwa | 10 | 2 | 15 | 0.1 | 88.2 | 99 | 16.7 | 0.7 | 10 | 15 | 0.1 | 0.1 | 0.7 | 99 | 60 | 50 |
| 500 | 211 | bowtie2 | 10 | 1 | 14 | 0.1 | 93.3 | 99 | 9.1 | 0.7 | 10 | 14 | 0.1 | 0.1 | 0.7 | 99 | 58.3 | 50 |
| 500 | 211 | bwa | 10 | 1 | 15 | 0.1 | 93.8 | 99 | 9.1 | 0.7 | 10 | 15 | 0.1 | 0.1 | 0.7 | 99 | 60 | 50 |
| 500 | 212 | bowtie2 | 10 | 1 | 15 | 0.1 | 93.8 | 99 | 9.1 | 0.7 | 10 | 15 | 0.1 | 0.1 | 0.7 | 99 | 60 | 50 |
| 500 | 212 | bwa | 10 | 1 | 18 | 0.1 | 94.7 | 99 | 9.1 | 0.6 | 10 | 18 | 0.1 | 0.1 | 0.6 | 99 | 64.3 | 50 |
| 500 | 213 | bowtie2 | 10 | 0.1 | 17 | 0.1 | 99.4 | 99 | 1 | 0.6 | 10 | 17 | 0.1 | 0.1 | 0.6 | 99 | 63 | 50 |
| 500 | 213 | bwa | 10 | 1 | 19 | 0.1 | 95 | 99 | 9.1 | 0.5 | 10 | 19 | 0.1 | 0.1 | 0.5 | 99 | 65.5 | 50 |
| 500 | 214 | bowtie2 | 10 | 1 | 13 | 0.1 | 92.9 | 99 | 9.1 | 0.8 | 10 | 13 | 0.1 | 0.1 | 0.8 | 99 | 56.5 | 50 |
| 500 | 214 | bwa | 10 | 1 | 22 | 0.1 | 95.7 | 99 | 9.1 | 0.5 | 10 | 22 | 0.1 | 0.1 | 0.5 | 99 | 68.8 | 50 |
| 500 | 215 | bowtie2 | 10 | 1 | 18 | 0.1 | 94.7 | 99 | 9.1 | 0.6 | 10 | 18 | 0.1 | 0.1 | 0.6 | 99 | 64.3 | 50 |
| 500 | 215 | bwa | 10 | 0.1 | 21 | 0.1 | 99.5 | 99 | 1 | 0.5 | 10 | 21 | 0.1 | 0.1 | 0.5 | 99 | 67.7 | 50 |
| 500 | 216 | bowtie2 | 10 | 0.1 | 20 | 0.1 | 99.5 | 99 | 1 | 0.5 | 10 | 20 | 0.1 | 0.1 | 0.5 | 99 | 66.7 | 50 |
| 500 | 216 | bwa | 10 | 2 | 17 | 0.1 | 89.5 | 99 | 16.7 | 0.6 | 10 | 17 | 0.1 | 0.1 | 0.6 | 99 | 63 | 50 |
| 500 | 217 | bowtie2 | 10 | 0.1 | 20 | 0.1 | 99.5 | 99 | 1 | 0.5 | 10 | 20 | 0.1 | 0.1 | 0.5 | 99 | 66.7 | 50 |
| 500 | 217 | bwa | 10 | 2 | 20 | 0.1 | 90.9 | 99 | 16.7 | 0.5 | 10 | 20 | 0.1 | 0.1 | 0.5 | 99 | 66.7 | 50 |
| 500 | 218 | bowtie2 | 10 | 0.1 | 17 | 0.1 | 99.4 | 99 | 1 | 0.6 | 10 | 17 | 0.1 | 0.1 | 0.6 | 99 | 63 | 50 |
| 500 | 218 | bwa | 10 | 1 | 19 | 0.1 | 95 | 99 | 9.1 | 0.5 | 10 | 19 | 0.1 | 0.1 | 0.5 | 99 | 65.5 | 50 |
| 500 | 219 | bowtie2 | 10 | 0.1 | 17 | 0.1 | 99.4 | 99 | 1 | 0.6 | 10 | 17 | 0.1 | 0.1 | 0.6 | 99 | 63 | 50 |
| 500 | 219 | bwa | 10 | 1 | 16 | 0.1 | 94.1 | 99 | 9.1 | 0.6 | 10 | 16 | 0.1 | 0.1 | 0.6 | 99 | 61.5 | 50 |
| 500 | 220 | bowtie2 | 10 | 1 | 12 | 0.1 | 92.3 | 99 | 9.1 | 0.8 | 10 | 12 | 0.1 | 0.1 | 0.8 | 99 | 54.5 | 50 |
| 500 | 220 | bwa | 10 | 1 | 15 | 0.1 | 93.8 | 99 | 9.1 | 0.7 | 10 | 15 | 0.1 | 0.1 | 0.7 | 99 | 60 | 50 |
| 500 | 221 | bowtie2 | 10 | 1 | 17 | 0.1 | 94.4 | 99 | 9.1 | 0.6 | 10 | 17 | 0.1 | 0.1 | 0.6 | 99 | 63 | 50 |
| 500 | 221 | bwa | 10 | 1 | 17 | 0.1 | 94.4 | 99 | 9.1 | 0.6 | 10 | 17 | 0.1 | 0.1 | 0.6 | 99 | 63 | 50 |
| 500 | 222 | bowtie2 | 10 | 0.1 | 15 | 0.1 | 99.3 | 99 | 1 | 0.7 | 10 | 15 | 0.1 | 0.1 | 0.7 | 99 | 60 | 50 |
| 500 | 222 | bwa | 10 | 0.1 | 20 | 0.1 | 99.5 | 99 | 1 | 0.5 | 10 | 20 | 0.1 | 0.1 | 0.5 | 99 | 66.7 | 50 |
