# Supplementary figures and images for: Identification of core and rare species in metagenome samples based on shotgun metagenomic sequencing, Fourier transforms and spectral comparisons
Source: ISME Commun. 2021 Mar 24;1:2. doi: 10.1038/s43705-021-00010-6 (PMC9645229; doi:10.1038/s43705-021-00010-6)

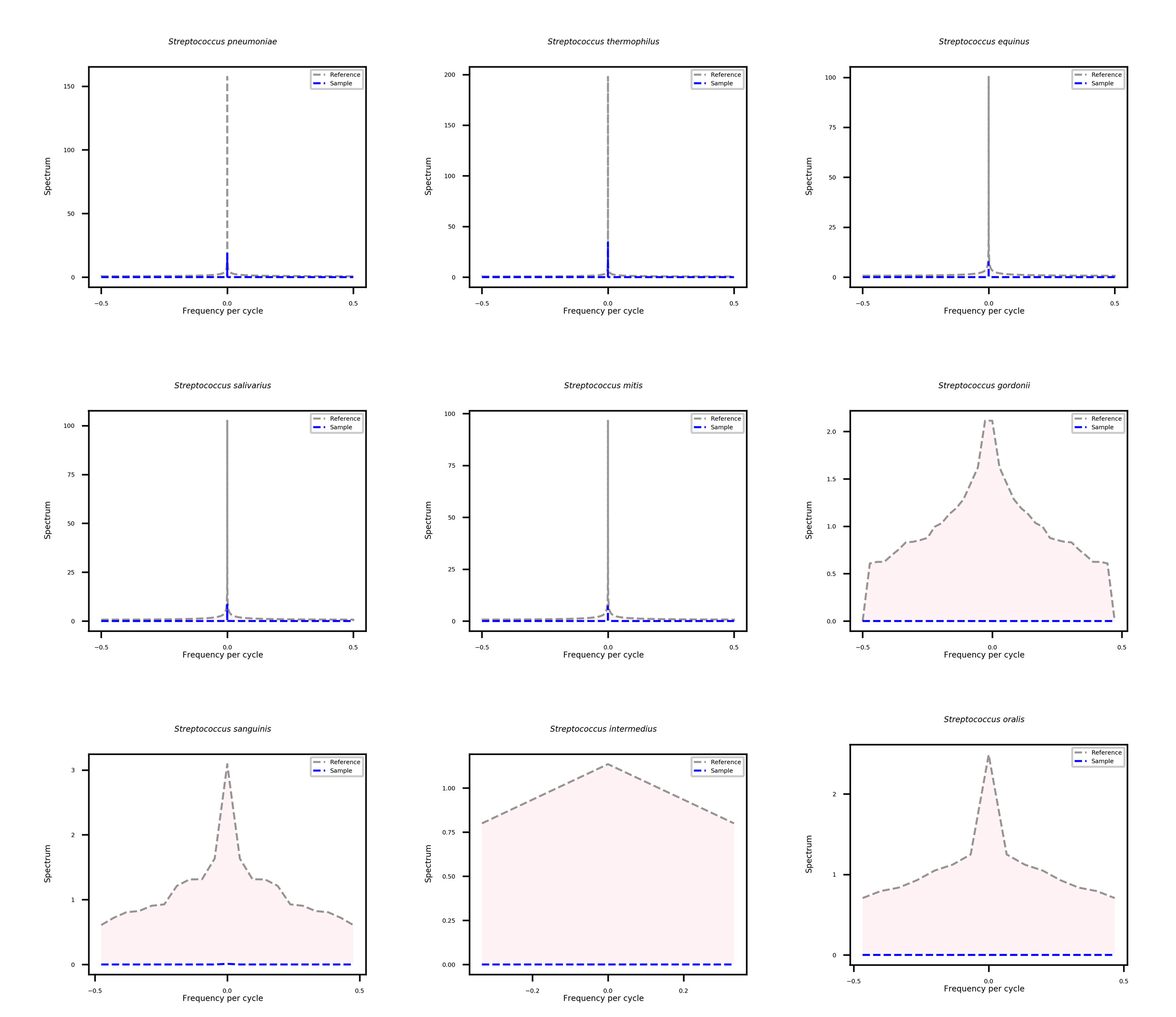

Supplement: Supplementary file 3 — Supplementary Figure 1 [file 43705_2021_10_MOESM3_ESM.tif]

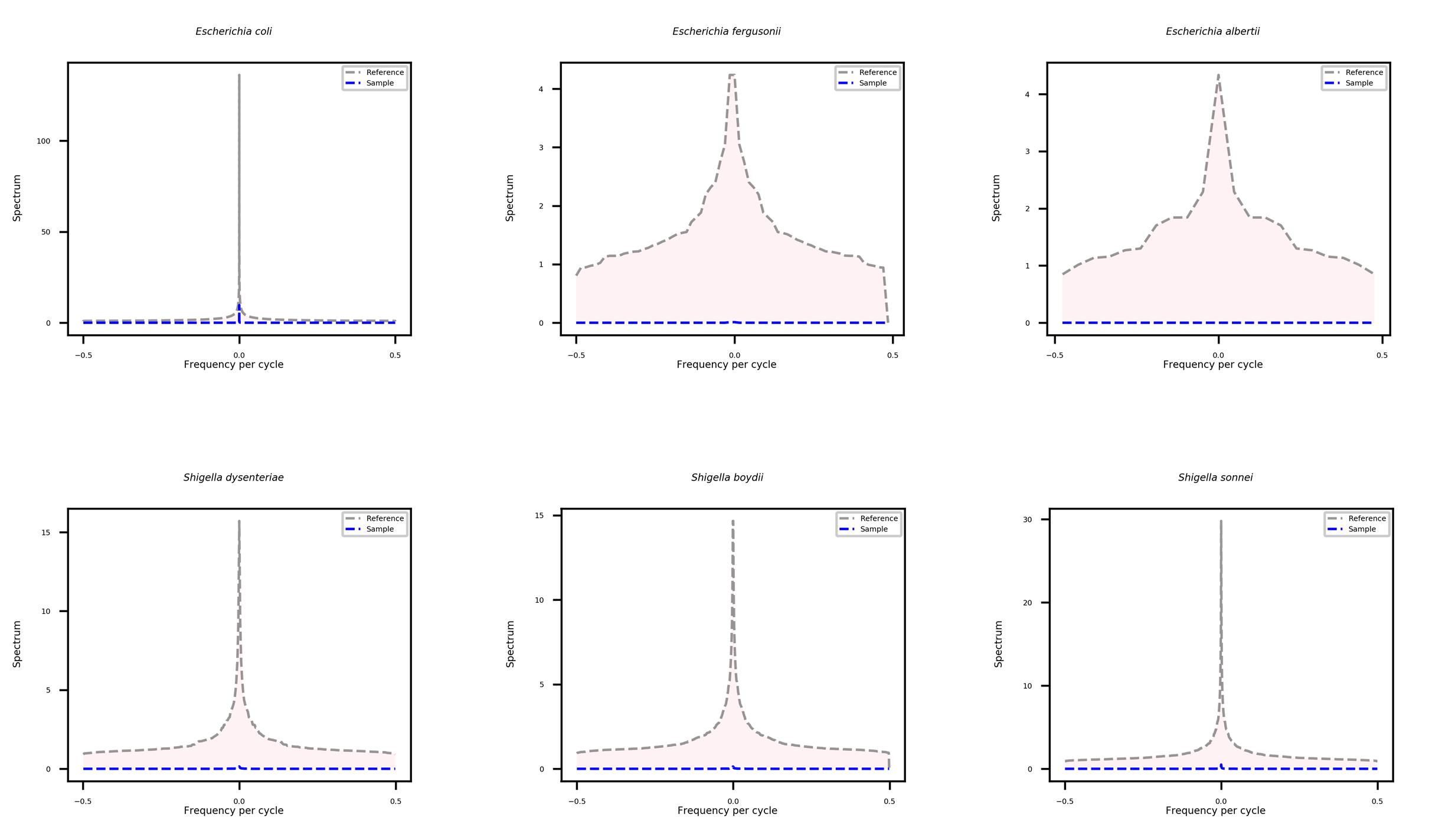

Supplement: Supplementary file 4 — Supplementary Figure 2 [file 43705_2021_10_MOESM4_ESM.tif]
