# Supplementary material for: Identification of core and rare species in metagenome samples based on shotgun metagenomic sequencing, Fourier transforms and spectral comparisons
Source: ISME Commun. 2021 Mar 24;1:2. doi: 10.1038/s43705-021-00010-6 (PMC9645229; doi:10.1038/s43705-021-00010-6)
Supplement: Supplementary file 8 — Supplementary Text 1 [file 43705_2021_10_MOESM8_ESM.pdf]

Supplementary text 1. Background information about the raspir algorithm

Discrete Fourier transforms are linear matrix-vector multiplications. During the mathematical operation, a vector x with discrete numerical data obtained from a function of time or a function of space is converted into a vector X with complex-valued frequency information (amplitude and phase) of signal x. Position-domain or time-domain functions describe how signals change in space or over time. Frequency-domain functions describe the refinements of wave-like signals by showing their energy distributions over a range of frequencies and hence unravel hidden patterns of the signal. The raspir tool builds two position-domain vectors x of equal length per species (Fig. 1a-b) The first reference vector x_R is a list of real-valued read distances x_{Ri} obtained from a simulated uniform distribution of short DNA reads across the reference genome (Fig. 1a).

$$x_R = \{x_{R0}, x_{R1}, x_{R2}, \ldots, x_{R(N-1)}\}$$

The second vector x_S comprises a list of real-valued distances of DNA reads obtained from a biological sample, which map towards a particular reference genome (Fig. 1b).

$$x_S = \{x_{S0}, x_{S1}, x_{S2}, \ldots, x_{S(N-1)}\}$$

To extract the underlying frequencies of the position domain input (x_R and x_S) and obtain the output vectors with complex Frequency coefficients of the reference (X_R, Fig. 1c) and biological sample (X_S, Fig. 1d), the input vectors are decomposed. So, if we have one input vector of length *N*, we generate *N* signals with one data point and each data point is multiplied by a Fourier matrix *M*.

$M = e^{-2\pi ink/N}$, where

N = total number of data points

2π / N = fundamental angular frequency

i = imaginary unit ($\sqrt{-1}$)

n = individual data point of the position-domain signal

k = frequency bin with values between 0 and N-1

The matrix *M* contains Euler's formula, which makes discrete Fourier transforms highly suitable for the application of evaluating distances of reads mapping towards circular bacterial genomes. While the position-domain vectors contain real numbers of read distances, Euler's formula enables us to work on a unit circle with cos x (real component) and sin x (imaginary components). So, we are again rotating along a circle for the subsequent mathematical transformation.

$E^{ix} = \cos x + i \sin x$ (Euler's formula)

If we now put both parts together, the position-domain vector and matrix *M*, we obtain the complete formula of the discrete Fourier transform, which is just the summation of *N* individual data points of the position-domain signal (x_n) multiplied by *M* to obtain the Fourier coefficient (X) at a frequency bin (k).

$$X_k = \sum_{n=0}^{N-1} x_n \times e^{-2\pi i n k/N} \qquad \text{for } 0 \leq k \leq N-1$$

If we first expand the summation for clarification and then abbreviate the equation, we obtain the complex Fourier coefficient (X) of a frequency bin (k) with its real (A) and imaginary (B) component. Finally, the absolute values (C_k) are obtained (magnitude spectrum) for the statistical comparison of reference and sample signals by applying Pearson's correlation and Euclidean distance measurements.

$$X_k = x_0[\cos(-\frac{2\pi nk}{N}) + i \sin(-\frac{2\pi nk}{N})] + \ldots + x_{(N-1)}[\cos(-\frac{2\pi nk}{N}) + i \sin(-\frac{2\pi nk}{N})]$$

$$X_k = A_k + i\, B_k$$

$$C_k = \sqrt{A_k^2 + B_k^2}$$

However, we have to consider that the input position-domain vectors can be very large, especially if we have many reads mapping towards a circular bacterial genome. With just ten short DNA reads, we have 45 distance combinations (N = 45). In case of 1 000 reads we are already left with 499 500 data points (N = 499 500). Therefore, in particular for core species the calculation becomes computationally very expensive. Plus, the algorithm of the discrete Fourier transform mentioned above, requires N^2 computational operations to

obtain the Fourier coefficients, because *N* sums are calculated and each sum has *N* components. Consequently, if we have 1 000 short reads mapping to just one bacterial genome, we undergo approximately 250 000 000 000 (= with $499\ 500^2$) computational operations for a species with poor genome coverage. As a consequence, we decided to implement a fast Fourier transform algorithm, which also computes the discrete Fourier transform but uses a computational complexity of N log(N) operations instead of N^2 operations.

This fast Fourier transform algorithm was introduced by Cooley and Tukey in 1965 [1]. Their basic idea was to perform the mathematical operation on sub-vectors of decreasing lengths. So, for a position signal with *N* data points, the first data point has index zero, the second data point has index one, and so on. The first sub-vector receives all data points with even indices and the second sub-vector contains all data points with odd indices. The discrete Fourier transform is subsequently performed on both of the sub-vectors separately. The two sub-vectors are further split into four shorter vectors with even and odd indices and the discrete Fourier transform is performed on these four sub-vectors as well. This is repeated until the input vector has two data points of indices zero and one. Whenever the input vector is broken down into sub-vectors with even and odd indices, the computational complexity of the discrete Fourier transform is reduced by a factor of two. So, with 1 000 short DNA reads, the fast Fourier transform requires about 3 000 operations, which is a major improvement in consideration of the approximately 250 000 000 000 computational operations with the standard algorithm of the discrete Fourier transform. Furthermore, we found that high-confidence species predictions are already feasible with less than 400 short reads. Therefore, if we have more than 400 reads aligning to a circular reference genome, raspir randomly selects 400 read positions and performs the discrete Fourier transform based on the subset of these read distances only. As a result, raspir requires about 1 000 operations per core species, less for rare species and hence runs on standard Desktop computers.

Further recommendations

Currently, a circular bacterial genome is a prerequisite for raspir. The reason for this is that usually the discrete Fourier transform treats the position-domain and the frequency-domain signal as periodic functions. So, if we have N reads aligning to a reference genome and we generate a position-domain signal based on

the reads' distance information, the discrete Fourier transform assumes that the N reads represent a single period of a periodic signal with infinite length. This means that our generated signal is connected to a second one, which is a duplicate of the first signal, and so on. If we have a circular genome, we can rotate along the circle endlessly long. Therefore, we meet the Fourier transform's requirement of a periodic signal and no matter how often we rotate along the genome, the generated time-domain signal of each rotation will perfectly fit into a single period. However, if we try to force a non-periodic linear chromosome with fixed end points into a discrete periodic frequency spectrum, the ends of the spectrum can extend into the following periods and distort the overall signal, because a linear construct cannot be directly integrated. In theory, it is possible to get the Fourier transform of read position combinations from linear chromosomes by convolving their position-domain signals with appropriate sinc functions. However, this is currently not addressed by raspir.

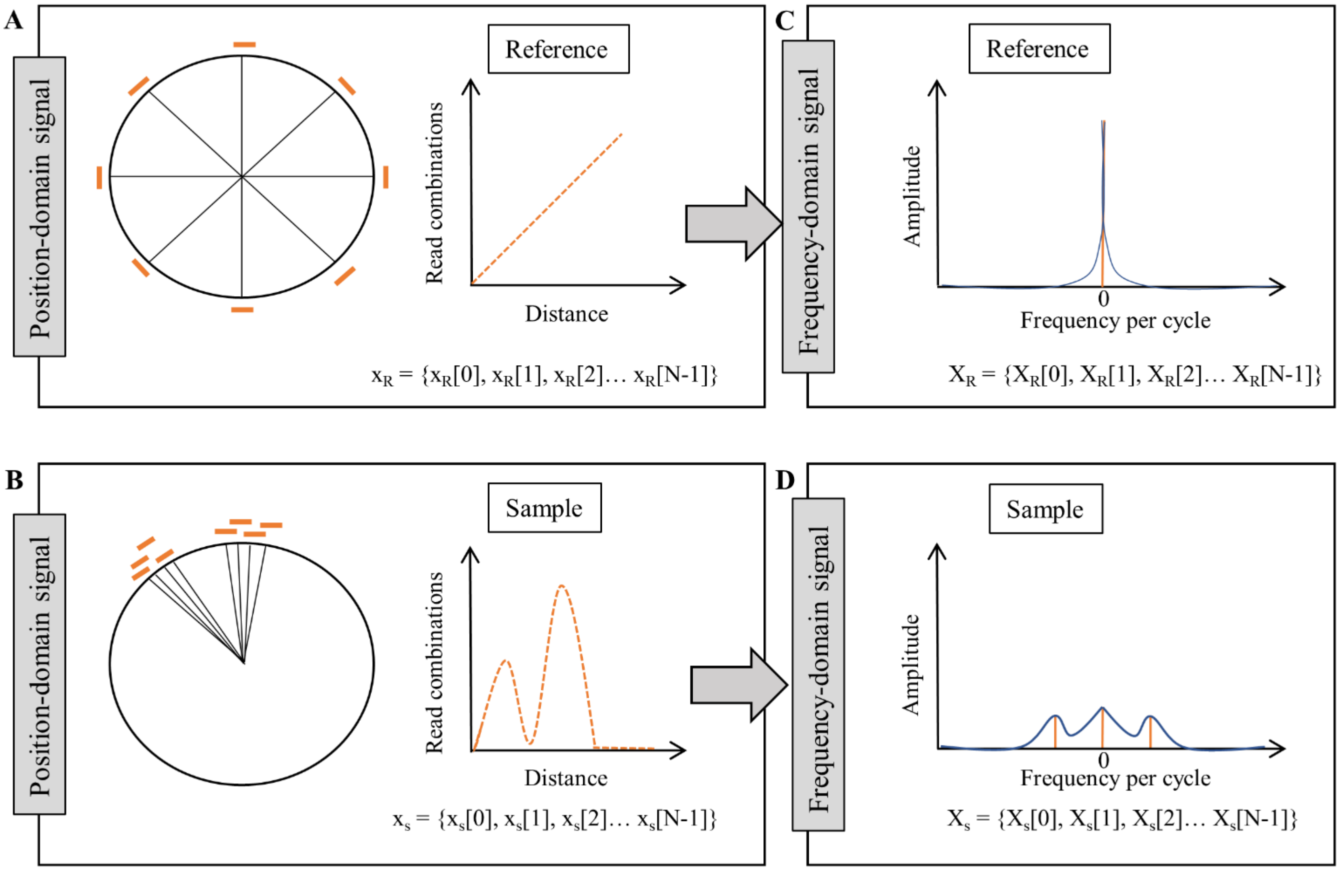

Figure 1. Simplified illustration of discrete Fourier transforms. (A) The reference's position-domain signal is generated from a simulated uniform read distribution. **(B)** The position-domain signal is obtained

from the real-world distribution of reads mapping to a reference genome. **(C)** The frequency-domain signal of a perfect uniform distribution is defined by a central, dominant base peak. **(D)** An example of a clustered read distribution represented as frequency-domain signal.

References

[1] Cooley JW, Tukey JW. An algorithm for the machine calculation of complex Fourier series. Math. Comput. 1965. Available from https://www.ams.org/journals/mcom/1965-19-090/S0025-5718-1965-0178586-1/S0025-5718-1965-0178586-1.pdf
