# Supplementary material for: Identification of core and rare species in metagenome samples based on shotgun metagenomic sequencing, Fourier transforms and spectral comparisons
Source: ISME Commun. 2021 Mar 24;1:2. doi: 10.1038/s43705-021-00010-6 (PMC9645229; doi:10.1038/s43705-021-00010-6)
Supplement: Supplementary file 9 — Supplementary legends [file 43705_2021_10_MOESM9_ESM.docx]

**Supplementary legends**

**Supplementary Table 1. Species selection and number of reads per species for eight simulation runs.** Simulation runs (1-8) were repeated with different seeds set for the ART Illumina read generator. Results for individual runs can be obtained from Supplementary Table 2.

**Supplementary Table 2.** Final result of the eight simulation runs with either 30, 50, 70, 100, 200, 300, 400 and 500 reads for the rare species. The eight simulation runs were repeated with twenty different seeds. The table shows raw count data for true positive (TP), false positive (FP), true negative (TN), false negative (FN) species. The specificity (true negative / (true negative + false positive)) and sensitivity (true positive / (true positive + false negative)), as well as the false discovery rate (FDR, 1 – (true positive / (true positive + false positive)) and the false omission rate (FOR, 1 – (true negative / (true negative + false negative)) are given as percentage values.

**Supplementary Text 1. Background information about the raspir algorithm**

**Supplementary Figure 1. The frequency-domain signals of *Streptococcus* spp. obtained from the representative simulation run seven.** The biological signal (blue) and the reference signal (black) of the true positive species *Streptococcus pneumoniae*, *Streptococcus thermophilus*, *Streptococcus equinus*, *Streptococcus salivarius* and *Streptococcus mitis* are significantly similar. The biological and reference signals of *Streptococcus gordonii*, *Streptococcus sanguinis*, *Streptococcus intermedius* and *Streptococcus oralis* are dissimilar. The distance between both signals is shown in light red.

**Supplementary Figure 2. The frequency-domain signals of *Escherichia coli* and *Shigella* spp. obtained from the representative simulation run seven.** The biological signal (blue) and the reference signal (black) of the true positive *Escherichia coli* are similar. The biological and reference signals of *Escherichia fergusonii*, *Escherichia albertii*, *Shigella dysenteriae*, *Shigella boydii* and *Shigella sonnei* are dissimilar. The distance between both signals is shown in light red.

**Supplementary Figure 3. Performance evaluation of raspir using publicly available real-world datasets.** From blank swabs (n = 5), maternal saliva samples (n = 11), foetal placental samples (n = 15) and maternal placental samples (n = 16), we randomly selected five patient samples and analysed them with raspir (first row) and without raspir (second row). The figure indicates presence/absence data of species per sample, where an organism is labelled as present if more than four reads mapped towards the corresponding reference genome. In the randomly selected maternal and foetal placental samples, only *Ralstonia pickettii* was detected by raspir.

**Supplementary Figure 4. Detection of *Ralstonia pickettii* across five randomly selected patient samples. (A)** *R. pickettii* was isolated from samples with varying sequencing depths. The sequencing depth ranged from 12 678 to 4 223 982 reads. **(B)** The number of raw reads mapping towards the *R. pickettii* reference genome was found to differ across samples, ranging from 85 raw reads to a maximum number of 1 267. The horizontal red line depicts the 100-read threshold.

**Supplementary Figure 5.** **Pairwise weighted Jaccard distances were calculated from normalised microbial community abundance data of maternal saliva samples.** The raw reads have been normalised for idealised genome length (to one million reference base pairs) and sequencing depth (to one million reads). The black colour depicts the inter-patient distance of microbial community composition obtained from datasets that were solely analysed without raspir (nraspir). The green colour depicts the pairwise intra-patient distance between datasets analysed with raspir versus without (raspir-nraspir). **(A)** Paired saliva samples of the same mother analysed with raspir and without (green) were significantly more similar in their microbial core community composition than saliva samples of different mothers (black). This was applicable for the 95 % of the most abundant species (Mann–Whitney U test, p-value < 0.0001, effect size r = 0.50, CI = 0.3 – 0.6), the 97 % of the most abundant species (Mann–Whitney U test, p-value < 0.0001, effect size r = 0.50, CI = 0.3 – 0.6) and the 99 % of the most abundant species (Mann–Whitney U test, p-value < 0.0001, effect size r = 0.48, CI = 0.3 – 0.6). **(B)** Paired saliva samples of the same mother analysed with raspir and without (green) were found to be significantly more distant in their rare microbial community composition than saliva samples of different mothers (black). This was applicable for the 1% of the least abundant species (Mann–Whitney U test, p-value < 0.0001, effect size r = 0.40, CI = 0.19 – 0.57), the 3 % of the least abundant species (Mann–Whitney U test, p-value < 0.001, effect size r = 0.30, CI = 0.05 – 0.55) and the 5 % of the least abundant species (Mann–Whitney U test, p-value < 0.01, effect size r = 0.23, CI = 0.08 – 0.48). Pairwise p values are given in the diagram with *p < 0.05, **p < 0.01, ***p < 0.001, ****p < 0.0001). The centre line of the boxplot depicts the median (50th percentile). The lower and upper boundary of the box represent the first (25th percentile) and third (75th percentile) quartile, and hence define the interquartile range (IQR). Whiskers extend from the box to the largest/smallest non-outlier data point (1.5 × IQR).
